# cellPLATO – an unsupervised method for identifying cell behaviour in heterogeneous cell trajectory data

**DOI:** 10.1242/jcs.261887

**Published:** 2024-06-12

**Authors:** Michael J. Shannon, Shira E. Eisman, Alan R. Lowe, Tyler F. W. Sloan, Emily M. Mace

**Affiliations:** ^1^ Department of Pediatrics, Vagelos College of Physicians and Surgeons, Columbia University Medical Center, NYC, NY 10032, USA; ^2^ Institute for the Physics of Living Systems, Institute for Structural and Molecular Biology and London Centre for Nanotechnology, University College London, London WC1H 0AH, UK; ^3^ Quorumetrix Solutions, Montreal, Québec H1T 2H5, Canada

**Keywords:** NK cells, Data-driven analysis, Unsupervised machine learning, Cell migration, Cell morphology, Phenomics, IL-15, Integrins

## Abstract

Advances in imaging, segmentation and tracking have led to the routine generation of large and complex microscopy datasets. New tools are required to process this ‘phenomics’ type data. Here, we present ‘Cell PLasticity Analysis Tool’ (cellPLATO), a Python-based analysis software designed for measurement and classification of cell behaviours based on clustering features of cell morphology and motility. Used after segmentation and tracking, the tool extracts features from each cell per timepoint, using them to segregate cells into dimensionally reduced behavioural subtypes. Resultant cell tracks describe a ‘behavioural ID’ at each timepoint, and similarity analysis allows the grouping of behavioural sequences into discrete trajectories with assigned IDs. Here, we use cellPLATO to investigate the role of IL-15 in modulating human natural killer (NK) cell migration on ICAM-1 or VCAM-1. We find eight behavioural subsets of NK cells based on their shape and migration dynamics between single timepoints, and four trajectories based on sequences of these behaviours over time. Therefore, by using cellPLATO, we show that IL-15 increases plasticity between cell migration behaviours and that different integrin ligands induce different forms of NK cell migration.

## Teaser

**Summary:** Cell plasticity and analysis tool (cellPLATO) is a new Python-based tool for phenotypic clustering using features of cell migration and morphology; here it is applied to the study of human lymphocytes.

## INTRODUCTION

Imaging populations of cells over time with microscopy reveals behavioural heterogeneity and plasticity, which is defined here as dynamic switching between behaviours over the course of observation. Immune cells function in a variety of environments, and their morphology and motility are intrinsic to these functions. Many immune cell functions rely on cells tuning their morphology and motility behaviour, which allows them to probe, migrate, kill and remember (Germain et al., 2012; Krummel et al., 2016; Lee and Mace, 2020, 2017). Advances in genetic and proteomic big data analysis techniques applied to biological data have enabled the stratification of subsets of cells using reduced dimensions. With the advent of fast, gentle imaging modalities, the development of bright cell labelling dyes, and the development of AI-based software for segmentation and tracking, similar analyses can be applied to features of cell images acquired using timelapse imaging. Here, we present a new Python-based tool, named the ‘Cell PLasticity Analysis Tool’ (cellPLATO; available at https://github.com/Michael-shannon/cellPLATO), which separates heterogeneous populations of migrating cells into behavioural subsets and analyses them through time. We apply cellPLATO to better understand the effect of IL-15 on natural killer (NK) cell migration on the integrin ligands intercellular adhesion molecule 1 (ICAM-1) and vascular cell adhesion molecule 1 (VCAM-1).

NK cell motility and changes in morphology are acquired through development and mediated by interactions with the microenvironment, including integrins and cytokine signalling (Björkström et al., 2016; Hegewisch-Solloa et al., 2021; Lee et al., 2020; Lee and Mace, 2020; Mace, 2023; Shannon and Mace, 2021). Lymphocyte function associated antigen 1 (LFA-1, the αLβ2 integrin) and very late antigen 4 (VLA-4, the α4β1 integrin) bind ICAM-1 and VCAM-1, respectively, and their balance tunes NK cell behaviour for crawling, diapedesis, tissue navigation and communication (Di Vito et al., 2019; Dominguez et al., 2015; Muller, 2009; Roy et al., 2020; Shannon et al., 2020; Valignat et al., 2013; Won Jun et al., 2022). The cytokine IL-15 is crucial for lymphocyte development (Huntington et al., 2008; Wang and Zhao, 2021; Wang et al., 2019) and integrin-mediated adhesion, migration and chemotaxis, but its biophysical role in acutely regulating crawling migration is understudied (Allavena et al., 1997; Lodolce et al., 1998; Nieto et al., 1996; Perera et al., 1999; Urlaub et al., 2017; Verbist and Klonowski, 2012).

Advances in fast, gentle, large field-of-view microscopy, combined with new methods for image segmentation (Lee et al., 2022; Pachitariu and Stringer, 2022; Stirling et al., 2021; Stringer et al., 2021; Tsai et al., 2019; Weigert et al., 2020) and cell tracking (Bove et al., 2017; Cuny et al., 2022; Ershov et al., 2022; Ulicna et al., 2021), have enabled the analysis of thousands of cells over time and brought microscopy imaging into the realm of big data. Existing tools highlight the importance of morpho-kinetic analysis and phenomics, allowing separation of cell behaviours, but often require intermediate coding knowledge to use and generate latent space behaviour categories without de-abstractification (Crainiciuc et al., 2022; Freckmann et al., 2022; Kimmel et al., 2021, 2018; Liu et al., 2023; Maity et al., 2022 preprint; Molina-Moreno et al., 2022; Wiggins et al., 2023). We sought to develop a tool to measure and classify cell populations downstream of such methods.

Here, we present a new software tool (cellPLATO) that separates heterogeneous populations of cells into instantaneous behavioural subsets followed by behavioural trajectory signatures over time. The tool (1) makes measurements of morphology, migration and clustering per cell per timepoint; (2) uses dimensionality reduction (UMAP) and cluster analysis (HDBSCAN) to designate instantaneous behavioural clusters; (3) uses sequence similarity, UMAP and HDBSCAN to designate behavioural trajectories over time; and then (4) generates data visualization including de-abstractification of cells within each cluster and fingerprinting to compare between conditions. To apply this tool, we enriched NK cells from human blood, treated them with IL-15, and measured characteristics of cell migration and morphology over time on ICAM-1 or VCAM-1 using cellPLATO. Together, our findings uncover novel aspects of NK cell biology and highlight the utility of phenomics-based analysis for understanding the heterogeneity of cellular populations.

## RESULTS

### Overview of cellPLATO workflow

**Fig. 1.**
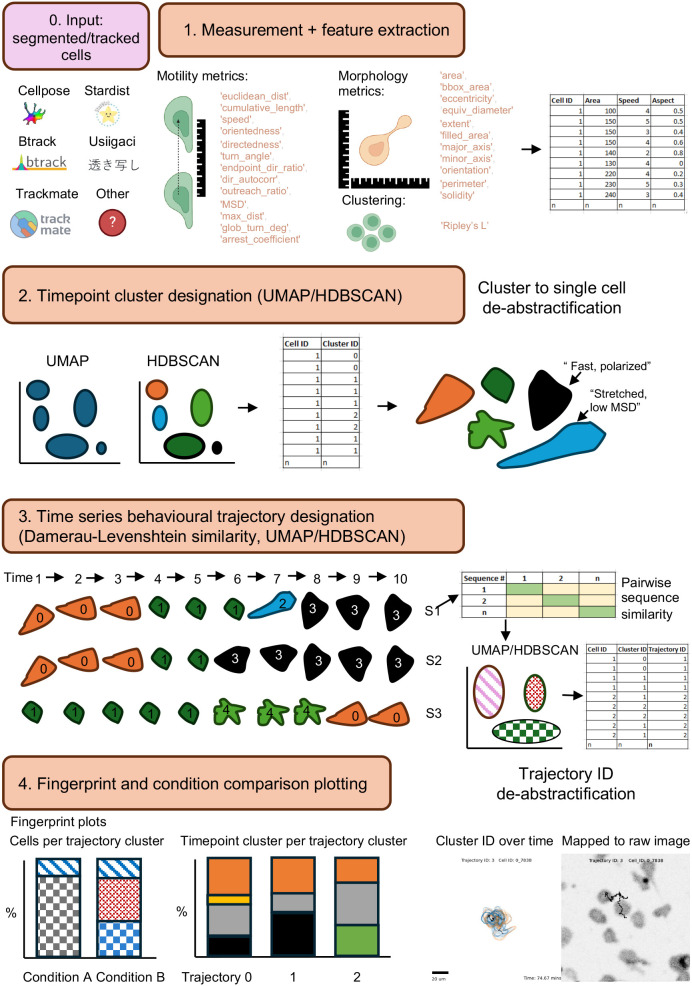
**Description of cellPLATO workflow.** The cellPLATO workflow consists of four steps hosted in a Jupyter notebook. Input of segmented cell data (step 0) precedes measurement of migration and morphology (step 1). Clusters based on these features are generated with UMAP and HDBSCAN (step 2) prior to generation of trajectories over time (step 3), which are visualized as frequency plots showing proportion of clusters per condition along with graphical de-abstractifications (step 4).

cellPLATO is executed in multiple steps broadly classified as (1) measurement, (2) UMAP and HDBSCAN timepoint behaviour cluster designation, (3) behavioural trajectory cluster designation, and (4) full visualization including de-abstractification of cells within each cluster and fingerprinting to compare conditions (Fig. 1). Briefly, .csv or h5 files containing tracking and segmentation data from btrack (Ulicna et al., 2021), Trackmate (Ershov et al., 2022) or Usiigaci (Tsai et al., 2019), are organized in a two-tiered hierarchy with separate folders for conditions at the top level and subfolders for experimental replicates within each condition. After collation of data from multiple conditions and replicates, cellPLATO calculates 28 migration and morphology metrics per timepoint per cell, then generates and saves plots of difference and plots of difference over time automatically for population averages of all metrics per condition. cellPLATO then uses UMAP and HDBSCAN, based on UMAP or n-dimensional data, to separate the cell population into subpopulations based on their morphological and motility-based features per timepoint, giving each cell at each timepoint a cluster ID. To explain the tangible nature of these clusters HDBSCAN identifies exemplars, which cellPLATO plots as graphics representing individual cells. The metrics that most contribute to the membership of each cluster based on their maximal variation from other clusters are listed next to each exemplar. To validate the data, clustering scores are extracted, and many exemplar cells from each cluster are visualized in a grid format. Having validated that single timepoint behaviour ID designations for every cell at every timepoint are reliable, the list over time provides a behavioural signature. cellPLATO uses Damerau–Levenshtein distance followed by a second layer of UMAP and HDBSCAN to group together similar behavioural sequences. Using this method, cellPLATO can identify subpopulations of changing cell behaviours over time, that is, behavioural trajectories. Trajectory cluster exemplars are extracted from the population data and displayed graphically, including as movies showing cluster change over time. These are coupled to static outputs to show the whole cell track and segmentation, as well as time plots of cluster membership. cellPLATO also generates a ‘fingerprint’ plot, representing percentage trajectory cluster distributions between conditions and defining the single timepoint cluster ID makeup of each trajectory ID. Coupled with graphical understanding of the nature of each trajectory, experimenters can intuitively understand which cell behaviours are the most and least prevalent in each condition. Finally, cellPLATO computes the cumulative plasticity of cells belonging to each trajectory ID, a measure of the number of single timepoint cluster switches performed by each cell over time. Taken together, cellPLATO offers a platform for comprehensively analysing heterogeneous segmentation and tracking data. The use of a single config file for input parameters simplifies analysis for those with limited coding experience, and the workflow unfolds automatically via modular Jupyter notebooks (https://jupyter.org/). The tool is optimized for handling large datasets, taking 7 h of computing time on a mid-range CPU to analyse the 47,815 cell tracks in this article, coupled with all visualization. The codebase itself is modular and adaptable should users wish to add new metrics prior to downstream analysis.

### NK cells have differing migration and morphology characteristics on ICAM-1 and VCAM-1

**Fig. 2.**
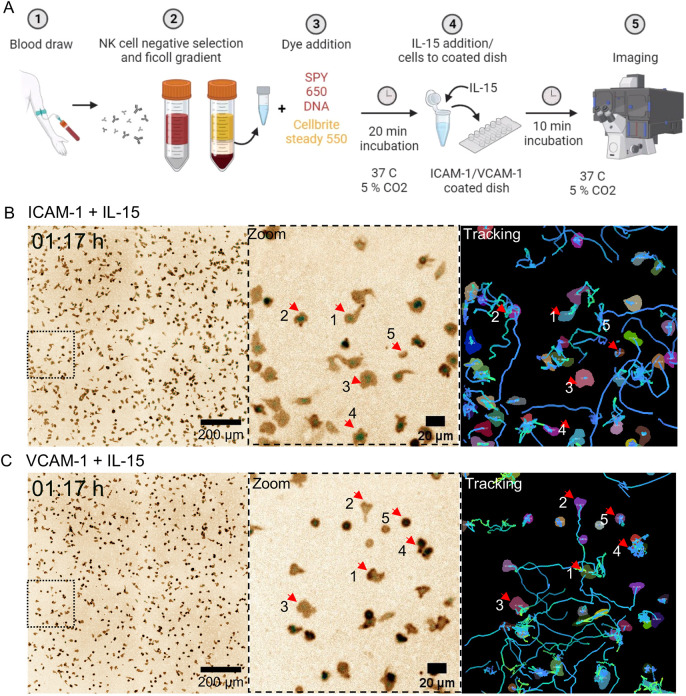
**Morphological and migratory features of NK cells imaged, segmented and tracked on ICAM-1 or VCAM-1 in the presence of IL-15.** (A) Freshly isolated human NK cells from Donor 1 labelled with membrane and DNA dye were imaged on ICAM-1 or VCAM-1 surfaces in the presence of IL-15. Timelapse images of NK cells on (B) ICAM-1 or (C) VCAM-1-coated glass. The dashed box (left) highlights the location of the magnified region (middle) and example cell segmentation and tracking (right). Red arrows denote different morphologies of cells identified qualitatively as described in the text. Representative images from 14,825 cells tracked from Donor 1. See also Movies 1 and 2.

To apply cellPLATO to study the migration characteristics of primary human NK cells, we isolated NK cells from peripheral blood from a healthy donor (Donor 1) using negative selection and confirmed by flow cytometry that >85% of the NK cells were positive for CD56 and negative for CD3, CD14 and CD19 (Fig. S1). Cells were labelled with DNA dye and membrane dye for 20 min before addition to an imaging chamber coated with ICAM-1 or VCAM-1 in medium containing IL-15. Timelapse confocal microscopy imaging began after a 10-min incubation (Fig. 2A). Following acquisition of imaging data, segmentation masks were generated with Cellpose (Stringer et al., 2021) and cells were tracked using btrack (Ulicna et al., 2021). Objects that were tracked for <8 frames (320 s, 5.3 min) or those with an area of <50 μm^2^ were filtered to exclude dead cells and debris. Cells were tracked for an average of 164 frames (6560 s, 109.3 min) and 14,825 distinct cell tracks were generated from Donor 1.

A diverse range of morphologies were identified qualitatively from the timelapse micrographs. NK cells on ICAM-1 exhibit polarized ‘teardrop’ shapes, semi-polarized shapes with wide lamellipodia, polarized shapes with multiple lamellipodia, or small and round shapes with no protruding edge (Fig. 2B, middle panel; red arrows 1 to 5). Qualitatively assessing cell tracks, we noted that teardrop-shaped cells moved for long distances, whereas cells with wider morphology moved for shorter distances. Multi-lamellipodial cells frequently changed direction, and small round cells did not migrate or began to migrate after a long period of quiescence (Fig. 2B, right panel; Movie 1 for raw timelapse; Movie 2 for segmentation and mask timelapse). Given that non-motile cells were stationary yet dynamic, we included them in our analysis. Qualitative assessment highlighted that cell morphology and migration coincided to produce different modes of behaviour. When comparing micrographs of NK cells on ICAM-1 with those on VCAM-1, we observed shared behaviours that appeared at different frequencies. Morphologically, NK cells on VCAM-1 appeared less spread than on ICAM-1, but readily migrated in the presence of IL-15 (Fig. 2C). Timelapse movies indicated that a subset of cells in the VCAM-1 condition were elevated at the uropod and lamellipodia, indicating adherence close to the back of the cell (Movies 1 and 2). The VCAM-1 condition generated small, rounded cells that appeared at a greater frequency than in the ICAM-1 condition (Fig. 2C). Thus, visual inspection of the micrographs, segmented masks and cell tracks suggested that subpopulations of NK cells reacted differently to ICAM-1 and VCAM-1 in the presence of IL-15 and that changes in morphology and motility should be considered together when defining cell behaviour.

### Rapid population-based changes in NK cell migration on ICAM-1 or VCAM-1 in the presence of IL-15

**Fig. 3.**
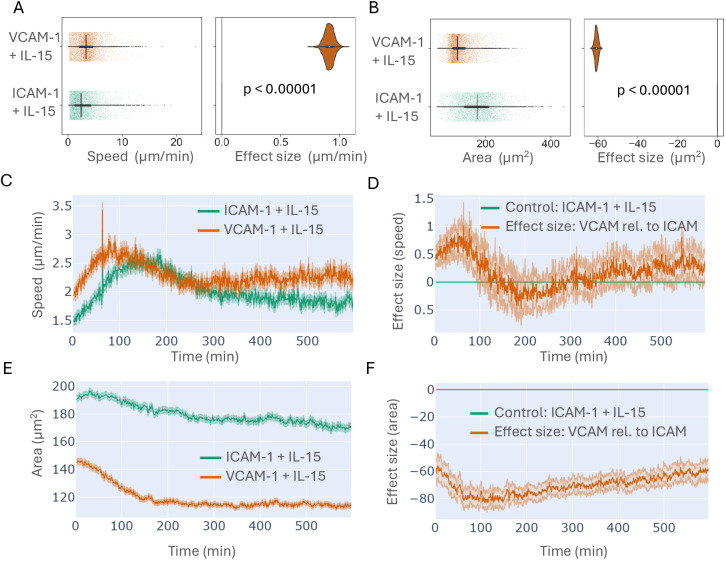
**Features of NK cell speed and cell area on VCAM-1 and ICAM-1 in the presence of IL-15.** Freshly isolated human NK cells from Donor 1 were imaged on ICAM-1 or VCAM-1 surfaces in the presence of IL-15. Cells were segmented and tracked as in Fig. 2 and features of cell shape and cell migration were calculated using cellPLATO. Plots of differences for (A) cell speed and (B) cell area show data distribution (left) and effect size (right). (C) Timeplot of mean cell speed on ICAM-1 or VCAM-1 in the presence of IL-15. (D) Timeplot of effect size for cell speed comparing VCAM-1 (orange line; VCAM-1+IL-15) relative to ICAM-1 control (green line; ICAM-1+IL-15). (E) Timeplot of mean cell area of cells on ICAM-1 or VCAM-1 in the presence of IL-15. (F) Timeplot of effect size for cell area comparing VCAM-1 (orange line; VCAM-1+IL-15) to ICAM-1 (green line; ICAM-1+IL-15). *n*=14,825 cells from Donor 1. A Kruskal–Wallis test was used to compare conditions and produce *P*-values. Plots in A and B display the distribution of the data as points (left) or a violin plot (right) showing the median (dot), interquartile range (IQR; thick line) and range (thin whiskers). Plots in C–F display the mean value per timepoint (line) and 95% c.i. (edge denoted by pale shades).

Having identified qualitative differences in cell morphologies between VCAM-1 and ICAM-1 conditions, we sought to understand how population-level metrics of cells were associated with single cell behaviours. cellPLATO makes 28 morphological and kinetic measurements per cell per timepoint calculated instantaneously or using time windows of, in this case, 320 s. This duration was used to optimize for the meaningful detection of cell behaviours while minimizing track length bias. cellPLATO then generates plots of difference (Goedhart, 2019 preprint) for each of the 28 morphological and kinetic metrics generated. Plots of difference are useful for large datasets where *P*-values become infinitesimally small due to sample size and do not necessarily reflect biologically relevant differences (Gómez-de-Mariscal et al., 2021). We first investigated two fundamental measurements of cell migration and morphology, namely cell speed and cell area. When comparing conditions, the median migration speed of NK cells was 3.48 μm/min on VCAM-1 and 2.54 μm/min on ICAM-1 (Fig. 3A). The effect size distribution for VCAM-1 did not overlap with the control condition (ICAM-1), suggesting statistical significance (Giavarina, 2015), which was confirmed by Kruskal–Wallis testing (*P*<0.00001). NK cells migrating on VCAM-1 also had a smaller median cell area (114 μm^2^) than those on ICAM-1 (175 μm^2^) (Fig. 3B), with a non-overlapping effect size distribution (*P*<0.00001 by Kruskal–Wallis testing).

The mean speed over time of cells migrating on ICAM-1 and VCAM-1 increased acutely following their contact with integrin ligands in the presence of IL-15. Cells on VCAM-1 reached a peak mean migration speed of 2.8 μm/min at 82 min before decreasing to a consistent 2.3 μm/min at 290 min until the end of imaging at 600 min (Fig. 3C). Cells on ICAM-1 took longer to reach a slightly lower peak mean migration speed of ∼2.55 μm/min at 120 min before decreasing to a plateau of <2 μm/min at 310 min (Fig. 3C). Visualization by plots of difference over time allowed us to statistically measure the difference over time between ICAM-1 as a reference condition and VCAM-1 by calculating and plotting the mean effect size at every time point. NK cells on VCAM-1 were significantly faster than ICAM-1 cells for the first 100 min reaching a peak mean difference of 1.02 µm/min, after which they were not statistically dissimilar (Fig. 3D).

Cell area was consistently higher over time in NK cells migrating on ICAM-1 compared with VCAM-1. NK cells on ICAM-1 had a continuous decrease in mean cell area beginning at 193 μm^2^ and ending at 174 μm^2^ (Fig. 3E). NK cells on VCAM-1 rapidly decreased in area in the first 150 min, beginning the experiment with a mean area of 142 μm^2^ and decreasing to a plateau of <120 μm^2^ at 150 min, through to the end of imaging. Plots of difference over time revealed a consistent statistically significant difference based on the non-overlap of the effect size distribution, where cells on VCAM-1 were ∼60 µm^2^ smaller relative to ICAM-1 throughout the experiment (Fig. 3F). Cells continued to migrate and change shape dynamically at the end of the experiment, indicating that observed changes were in response to ICAM-1 or VCAM-1 and not phototoxicity during imaging.

In summary, we observed an acute cell speed increase in NK cells migrating on VCAM-1 or ICAM-1 in the presence of IL-15, where cells on VCAM-1 reached a maximum more rapidly. Cells on ICAM-1 had consistently larger cell areas than cells on VCAM-1, which experienced a significant decrease in cell area in the first 150 min of the experiment. Together these data demonstrate that NK cells have different migratory features in response to IL-15 that are dependent on integrin ligands.

### NK cells migrating on ICAM-1 or VCAM-1 can be grouped into eight distinct per-timepoint behavioural clusters using UMAP and HDBSCAN

Although population-based averages identified differences in cell speed and area, they did not stratify subpopulations of cells responding differently to IL-15, ICAM-1 or VCAM-1. Single metrics cannot capture the complex nature of single-cell behaviour, so we sought to group cells based on multiple metrics measured between adjacent timepoints. cellPLATO performs UMAP on morphological and motility parameters, and HDBSCAN cluster analysis to define behavioural clusters. Exemplar cells that best represent each cluster are displayed as single cell graphics to de-abstractify the behaviour that each cluster represents, and top contributing metrics are displayed for each behaviour type.

**Fig. 4.**
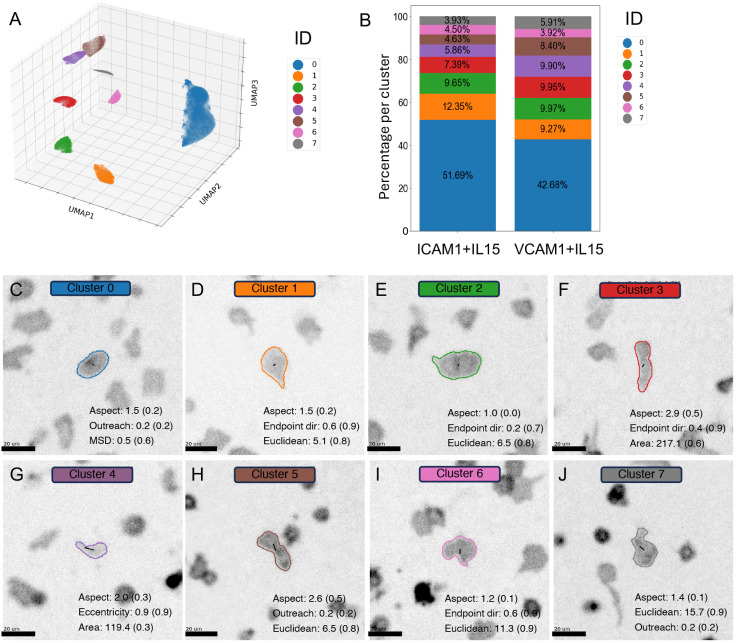
**Distinct behaviours de-abstractified from NK cells migrating on ICAM-1 or VCAM-1 in the presence of IL-15.** (A) Eight clusters of distinct cell behaviours based on morphological and motility-based metrics identified by UMAP followed by HDBSCAN cluster analysis. (B) Percentage cluster ID by condition. (C–J) Exemplar cells representing each cluster shown as cell contours and tracks overlaid onto raw data accompanied by the metrics that best define membership in that cluster (scaled values in parentheses). *n*=14,825 cells from Donor 1.

We performed dimensionality reduction and cluster analysis on our combined dataset of NK cells migrating on VCAM-1 or ICAM-1 after IL-15 activation, and plotted UMAPs 1, 2 and 3. Each point represents the behavioural status of a single cell at each timepoint, and cells belonging to clusters identified using HDBSCAN are differentiated by colour (Fig. 4A). The cluster ID defined by UMAP and HDBSCAN represents the instantaneous morpho-kinetic state of a cell in relation to the range of cell behaviours observed over the course of the experiment. In our dataset, eight distinct clusters of cells exhibiting distinct behaviours were identified over the range of UMAP and HDBSCAN hyperparameters tested. To understand the distribution of these per timepoint cell migration behaviours on VCAM-1 or ICAM-1, the percentage of cells found in each behavioural cluster was calculated to produce a behavioural ‘fingerprint’ for each condition (Fig. 4B).

To explore the quality of clustering, we calculated a silhouette score (see Materials and Methods) of 0.84 (range: −1 to 1). cellPLATO gives users the option to output several cluster scoring metrics, but such scoring methods are highly dependent on the nature of the data. We therefore combined scoring the clusters with visualization of cell contours from each cluster and the top three contributory metrics to their cluster membership (Fig. S2). Cells belonging to the same cluster owing to their proximity in dimensionally reduced space were highly visually similar and shared similar morphological and migratory characteristics. As density-based clustering identifies areas of varying density and groups together points in high-density regions, a data point might be considered unclustered when it resides in a region of low density (an extreme outlier) or between two areas of high density (boundary outlier). In this analysis, no datapoints were assigned as unclustered. We also visualized the contribution of each metric as a heat map in UMAP space (Fig. S3). Metrics contributed to different extents to different clusters, indicating that they were useful in separating cells into behavioural subsets.

To intuitively display cell migration and morphology behaviours, cellPLATO graphically displays exemplar cells from each cluster, optionally overlaid on microscopy images, and their three most contributory metrics that define cluster membership (Fig. 4C–J). Cells in clusters had a range of behaviours driven by distinctive combinations of aspect ratio, endpoint directionality, outreach ratio, mean squared displacement (MSD), eccentricity and area. These de-abstractified per timepoint cell migration behaviours are colour-matched to the behavioural ‘fingerprint’ plot for each condition; using this analysis, clear proportional differences in the representation of the eight behaviours emerged when comparing NK cells interacting with VCAM-1 compared with ICAM-1 (Fig. 4B). NK cells from ICAM-1 or VCAM-1 conditions occupied six of the eight clusters in different proportions, and cluster IDs 2 and 6 in almost the same proportion (<0.6% difference in these cluster proportions between conditions; Fig. S4). To complement the graphics and the fingerprint plot, the defining characteristics for each cluster are described in Table S1, and median values are available in Table S2. Considering the frequencies of cells in each cluster, our results suggest that ICAM-1 induced a phenotype defined by medium polarization, medium displacement and directed medium to fast migration, whereas VCAM-1 induced more extreme polarization and faster movement, with cells more frequently returning close to their initial position.

### Behavioural sequence analysis reveals stationary, confined, oscillatory and exploratory sub-populations of cell behavioural trajectories through time

**Fig. 5.**
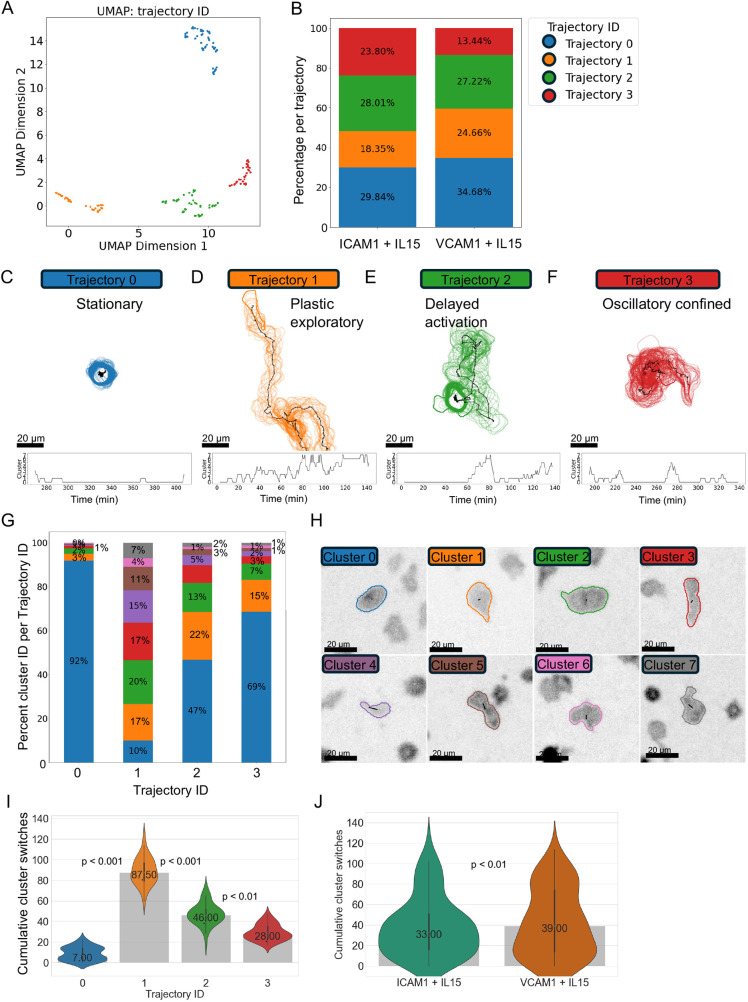
**Behavioural trajectory cluster analysis reveals differential trajectories of NK cells migrating on ICAM-1 or VCAM-1.** Trajectory analysis of NK cells from Donor 1 generated by pairwise similarity comparison of cluster ID time sequences. (A) UMAP plot coloured by trajectory ID. (B) Fingerprint plot showing the percentage trajectory ID per condition. (C–F) Graphical representations (top) and time plots of behavioural cluster ID (bottom) for each trajectory ID. See also Movies 3–6. (G) Fingerprint plot showing the frequency of single timepoint behavioural cluster IDs for each trajectory ID. (H) Graphical depictions of single timepoint behaviours represented in G and reproduced from Fig. 4C–J. (I) Plasticity of cells per behavioural trajectory ID measured by the median number of cluster switches. (J) Median number of cluster switches per condition. *n*=187 cells filtered to include only cells with 200 to 220 timepoints randomly selected from 14,825 cells from Donor 1. Kruskal–Wallis followed by Wilcoxon rank-sum testing with Bonferroni correction was used to compare between trajectories (I) or conditions (J); *P*<0.00001 for all pairwise comparisons. The data distribution is shown as a violin plot with median (dot, printed value), interquartile range (IQR; thick line) and range (thin whiskers).

To see how behaviours of cells at single timepoints correlated with their behaviour over time, we performed behavioural trajectory analysis. Every cell has a behavioural signature at each timepoint defined by their cluster ID. Therefore, single cell tracks can also be converted into a categorical number sequence reflecting changes in behaviour over time as they switch between cluster IDs. cellPLATO implements sequence similarity analysis using Damerau–Levenshtein (normalized edit distance) (Damerau, 1964) to perform pairwise comparison of trajectories of cell tracks as they change over time. Similarity scores are used with UMAP and HDBSCAN to group sequences of similar sequences and to designate each type with a ‘trajectory ID’. For clarity, whereas the ‘behavioural clusters’ identified previously are an indicator of the behavioural status of a cell at a given time, the trajectory IDs are groupings of cells with similar behavioural sequences through time. Although normalization helps reduce the influence of sequence length, sequences of vastly different length cannot be meaningfully compared by edit distance metrics. Therefore, analysis was performed on a filtered subset of 187 cells (ICAM-1, 104; VCAM-1, 83) with similar track lengths in time (200–220 timepoints) from Donor 1. We confirmed that this dataset maintained the same behavioural trends between conditions to that in the full dataset (Fig. S5). Sequence similarity analysis identified four distinct behavioural trajectories with clear separation in UMAP space (Fig. 5A). Measuring the distribution of trajectories per condition showed that trajectories were found in different frequencies when comparing NK cells on ICAM-1 versus VCAM-1 (Fig. 5B). Specifically, the proportion of trajectory 0 and trajectory 1 cells was reduced on ICAM-1 compared with VCAM-1 (−4.84% and −6.31%, respectively; Fig. 5B, blue and orange bars) whereas trajectories 2 and 3 were increased (by 0.79% and 10.36%, respectively; Fig. 5B, green and red bars). Trajectories 1, 2 and 3 represent motile cells, whereas trajectory 0 is composed of stationary cells (Fig. 5C–F). The summed percentage membership of motile trajectories 1–3 are 70.14% for ICAM-1+IL-15 and 65.32% for VCAM-1+IL-15. This difference suggests that, while motile behaviours are slightly increased on ICAM-1, differences are greater between the frequencies of trajectories representing different motile behaviours, particularly trajectory 3, when comparing between the two conditions.

**Fig. 6.**
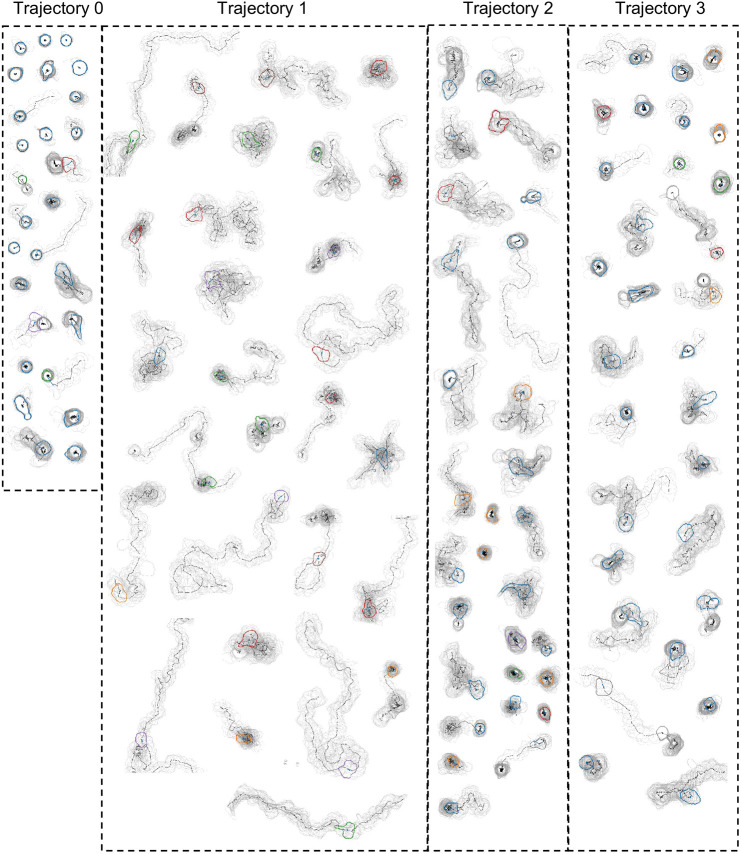
**Gallery of behavioural trajectory IDs as contour plots and time traces.** Trajectory analysis was performed to group cells based on their morphological and migratory behaviour over time. Shown are representative cells assigned to each trajectory ID. *n*=187 cells from Donor 1.

To understand the behavioural nature of each trajectory type, we de-abstractified the trajectory ID clusters by generating exemplar trajectories. These were displayed as graphics denoting their contour and track through time coloured by trajectory ID; under each example is a plot of their single timepoint cluster ID over time (Fig. 5C–F). To further ratify the graphical exemplars shown, a gallery of examples of each trajectory (Fig. 6) and their cluster ID over time (Fig. S6) were plotted; their similarity described with a silhouette score of 0.76.

To quantify the behaviours making up cells belonging to each trajectory ID, the composition of each trajectory ID by single timepoint cluster ID was plotted (Fig. 5G). The single timepoint exemplar cell graphics representative of each of the single timepoint cluster IDs from Fig. 4 are shown in Fig. 5H and are described in Table S1. Together, the plot and graphics show that each trajectory ID is made up of different proportions of identified single timepoint behaviours (Fig. 5G,H). The fingerprint of single timepoint behaviours defines the composition of each trajectory ID (Fig. 5G) but not the frequency by which cells switch between clusters. To quantify this, we calculated the number of single timepoint cluster ID switches made by each cell (Fig. 5I). Trajectory 0 cells make a median of 7 switches over their time course (200 to 220 frames; 133 to 146 min), trajectory 1 cells make 87.5 switches, trajectory 2 cells make 46 and trajectory 3 cells make 26 (Fig. 5I). Together, this indicates that the trajectories we identified have distinct behavioural plasticity. We then compared cluster switching between cells on ICAM-1 versus VCAM-1 in the presence of IL-15, independently of identified trajectory IDs, where VCAM-1 induced significantly more switching between clusters than ICAM-1 (Fig. 5J).

In summary, trajectory analysis revealed four subpopulations of cells identified by clustering similar strings of single timepoint behaviours (cluster IDs) in feature space, namely a stationary subset (trajectory 0; Movie 3), a highly plastic exploratory subset (trajectory 1; Movie 4), a delayed activation subset (trajectory 2; Movie 5), and an oscillatory confined migration subset (trajectory 3; Movie 6). All trajectories were represented on ICAM-1 and VCAM-1 surfaces. However, on ICAM-1, the oscillatory confined migration subtype (trajectory 3) was much more prevalent than on VCAM-1, where the highly plastic and exploratory subset (trajectory 1) was more highly represented.

### IL-15 acutely changes the migration and morphology of NK cells on integrin ligands

Given the highly migratory behaviour of NK cells migrating on ICAM-1 or VCAM-1 in the presence of IL-15, we wanted to determine the effect of IL-15 on cell migration behaviour relative to integrin ligands alone. Having analysed cells from a single blood donor (Donor 1), we also sought to analyse additional biological replicates using independent clustering and analysis.

We isolated NK cells from two additional blood donors (Donors 2 and 3) and imaged cells migrating in the presence of IL-15. As an additional comparison, we included conditions from Donor 3 in which cells were incubated on ICAM-1 or VCAM-1 in the absence of IL-15. We then performed cellPLATO analysis on the data acquired from Donors 2 and 3. Cell segmentation and tracking were performed using the same approaches as our previous dataset. Measurement of cell migration and morphology characteristics at each timepoint for each of the four conditions from the two donors was performed, generating 32,990 cell tracks. Specifically, Donor 2 had 3150 cells (ICAM-1+IL-15, 1291; VCAM-1+IL-15, 1859) and Donor 3 had 29,840 cells (ICAM-1, 5207; ICAM-1+IL-15, 8447; VCAM-1, 9575; VCAM-1+IL-15=6611).

**Fig. 7.**
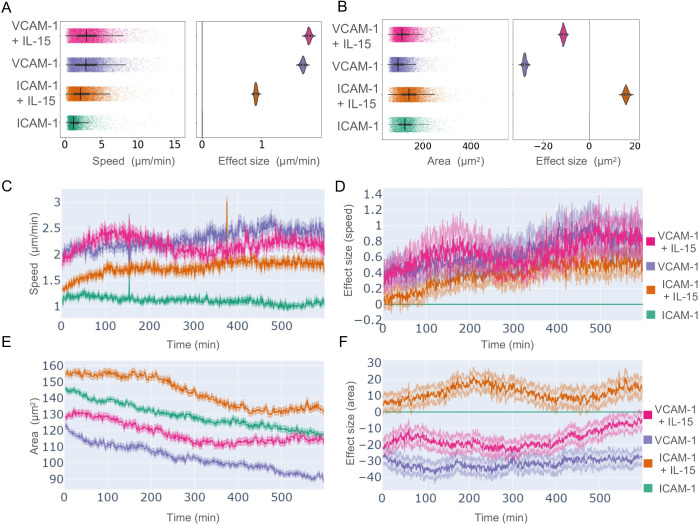
**Population behaviours of NK cells migrating on ICAM-1 or VCAM-1 in the absence and presence of IL-15.** Freshly isolated human NK cells from two healthy donors were imaged on ICAM-1 or VCAM-1 surfaces in the presence of IL-15 (Donor 2) or the absence or presence of IL-15 (Donor 3). Cells were segmented and tracked, and features of cell shape and migration calculated. Plots of differences for (A) cell speed and (B) cell area show data distribution (left) and effect size (right). (C) Timeplot of mean cell speed. (D) Timeplot of effect size for cell speed on VCAM-1+IL-15 (pink line), VCAM-1 (purple line), ICAM-1+IL-15 (orange line) relative to ICAM-1 control (green line; ICAM-1). (E) Timeplot of mean cell area over time. (F) Timeplot of effect size for cell area on VCAM-1+IL-15 (pink line), VCAM-1 (purple line), ICAM-1+IL-15 (orange line) relative to control (green line; ICAM-1). *n*=32,990 cells from Donor 2 (3150 cells) and Donor 3 (29,840 cells); conditions with IL-15 are combined from both donors and conditions in the absence of IL-15 are Donor 3 only. Kruskal–Wallis followed by Wilcoxon rank-sum testing with Bonferroni testing was used to compare all conditions; *P*<0.0001 for all pairwise comparisons. Plots A and B display the distribution of the data as points (left) or a violin plot (right) showing the median (dot), IQR (thick line) and range (thin whiskers). Plots C–F display the mean value per timepoint (line) and 95% c.i. (edge denoted by pale shades). Donor 2 and 3 experiments were performed independently.

To understand the global effect of IL-15 on cell migration, we plotted the median speed, including effect size, of NK cells migrating on ICAM-1 or VCAM-1 in the presence of IL-15 (Donors 2 and 3) or in the absence of IL-15 (Donor 3). NK cells on ICAM-1 in the absence of IL-15 had a lower median speed (1.17 µm/min) compared with those on VCAM-1 in the absence of IL-15 (2.87 µm/min) (Fig. 7A). Consistent with our previous observations from Donor 1 (Fig. 3C), NK cells from Donors 2 and 3 on ICAM-1 in the presence of IL-15 had a median speed of 2.07 µm/min, whereas those on VCAM-1 in the presence of IL-15 had a median speed of 2.97 µm/min (Fig. 7A). The addition of IL-15 increased the cell area of cells migrating on either ICAM-1 or VCAM-1; however, cells on ICAM-1 had greater area than those on VCAM-1 (Fig. 7B). Over time IL-15 produced an acute effect of greater speed in cells on ICAM-1, which then reached a consistent maximum (Fig. 7C; effect size shown in Fig. 7D). We observed a similar acute effect of IL-15 on cells on VCAM-1, although this was more variable and the difference between VCAM-1 alone and VCAM-1+IL-15 at the end of the time of imaging was minimal. The area of cells in all conditions slowly decreased over time (Fig. 7E; effect size shown in Fig. 7F) and we similarly observed decreased cell area of cells from Donors 2 and 3 on VCAM-1 conditions as observed for Donor 1 in Fig. 3. Finally, to directly compare inter-donor variability we plotted the speed and area of cells from each donor migrating in the presence of IL-15 (Fig. S7). Although donor-to-donor differences were observed, previously noted trends were consistent between all three donors for speed (Fig. S7A) and area (Fig. S7B). Despite similar median values, statistical comparisons of speed and area were significantly different between donors, likely due in part to the large number of datapoints included in our analysis. This result underscores the utility of considering effect sizes in addition to *P*-values when attempting to compare large datasets of biological data.

**Fig. 8.**
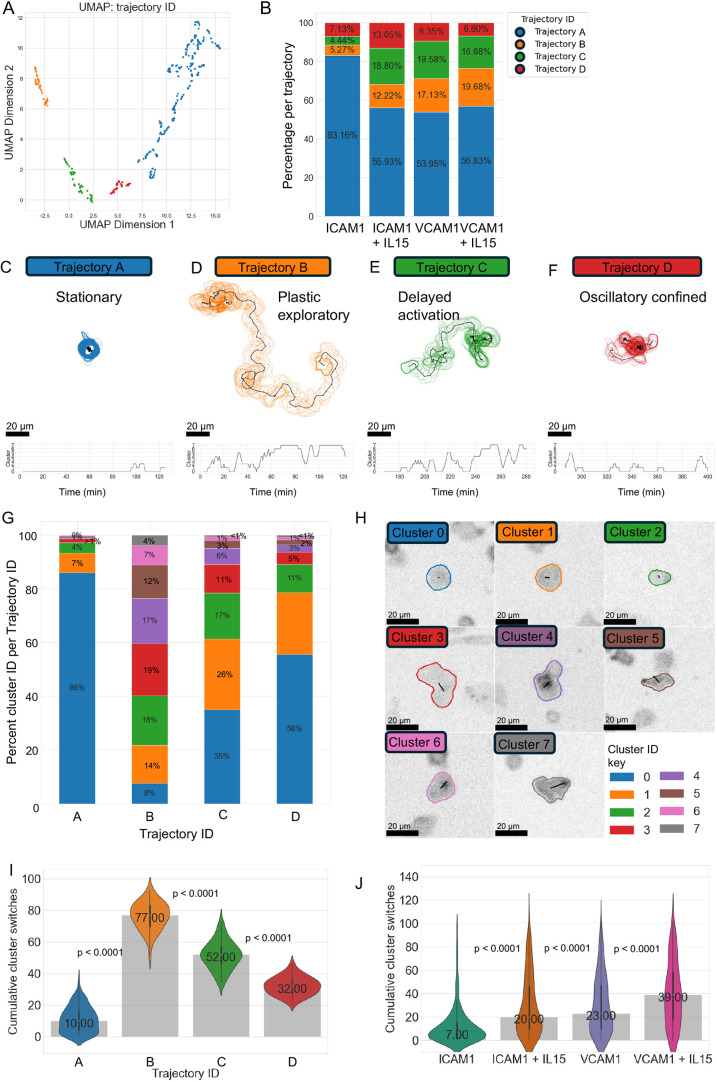
**Effect of IL-15 on behaviourally distinct NK cell subsets migrating on ICAM-1 or VCAM-1.** (A) UMAP plot of data from Donors 2 and 3 in the absence (Donor 3) or presence and absence of IL-15 (Donors 2 and 3) coloured by trajectory ID. (B) Fingerprint plot showing the percentage of each trajectory ID per condition. (C–F) Graphical representations for trajectories A to D (top) and timeplots of single timepoint cluster ID (bottom). (G) Fingerprint plot showing the distribution of single timepoint behavioural cluster IDs for each trajectory ID. (H) Graphical depictions of single timepoint behaviours represented in G overlaid on raw data. (I) Plasticity of cells in each trajectory ID measured by the median number of cluster switches. (J) Cumulative cluster switches between conditions. *n*=411 cells filtered to include only cells with 200 to 220 timepoints randomly selected from 32,142 cells from Donors 2 and 3 (IL-15 conditions) or Donor 3 (no IL-15 condition). Kruskal–Wallis followed by Wilcoxon rank-sum testing with Bonferroni correction was used to compare between trajectories (I) or conditions (J); *P*<0.00001 for all pairwise comparisons. Data distribution shown as a violin plot with median (dot, printed value), IQR (thick line) and range (thin whiskers). Donor 2 and 3 experiments were performed independently.

Following population analysis, we continued with the cellPLATO workflow of cells migrating in presence of IL-15 (Donors 2 and 3) or absence of IL-15 (Donor 3). We again identified eight single timepoint behaviour clusters, and subsequently designated trajectory IDs from single timepoint cluster IDs as shown above in Fig. 5. Data were filtered to include only track lengths of 200 to 220 timepoints before plotting their UMAP coordinates coloured by trajectory ID, which demonstrated four well-separated trajectory clusters (Fig. 8A) with differential representation between experimental conditions (Fig. 8B). In this analysis, four cells of 411 total were classified as unclustered, and so were removed from further analysis.

As clusters and trajectories from Donors 2 and 3 were calculated in a separate latent space to those of Donor 1, we named them trajectories A, B, C and D (Fig. 8) to differentiate them from trajectories 0, 1, 2 and 3 shown in Fig. 5. Representative graphics allowed us to visualize the phenotypes of trajectories A–D and compare them with previously identified trajectories 0–3. Despite the small differences in cell speed and area measured in Fig. S7, trajectory cluster-based cell behaviours were conserved between Donor 1 (Fig. 5) and Donors 2 and 3 (Fig. 8). Although the proportion of each trajectory was different (Fig. 8B versus Fig.5B), trends between conditions were maintained. We manually aligned the trajectories A–D with 0–3; specifically, trajectory A (Fig. 8C) had similar stationary properties to trajectory 0 (Fig. 5C), trajectory B (Fig. 8D) had similar exploratory properties to trajectory 1 (Fig. 5D), trajectory A (Fig. 8E) had similar properties of delayed migration to trajectory 2 (Fig. 5E), and trajectory D (Fig. 8F) had similar oscillatory confined properties to trajectory 3 (Fig. 5F). We then calculated the proportion of each single timepoint cluster ID for each of the four trajectories (Fig. 8G) and visualized graphical exemplar representations of the single timepoint cluster IDs (Fig. 8H).

Finally, we compared the number of cluster switches each cell made over the 200 to 220 timepoints of imaging used to generate trajectories (Fig. 8I). As previously observed for analogous trajectories in Fig. 5, trajectory A cells were the least behaviourally plastic, making a median of 10 behavioural switches, followed by trajectory B cells making a median of 77 switches, trajectory C cells making a median of 52 switches and trajectory D cells making a median of 32 switches. Independently of trajectory IDs, we compared cluster switching between conditions, and found that the presence of IL-15 increased the number of times cells switched clusters on both ICAM-1 and VCAM-1, with cells on VCAM-1 switching clusters more frequently than their counterparts on ICAM-1 in the absence of IL-15 (Fig. 8J).

Together, these analyses show that a switch to more plastic migratory behaviour happens in direct response to IL-15 addition. Furthermore, we confirmed that the four different modes of migratory and morphological behaviour that we previously identified in a single donor are consistently present in NK cells isolated from human blood. These include a stationary mode that is unresponsive to IL-15, a mode with delayed activation, a mode that is highly exploratory, and a mode that oscillates between short bursts of migration and arrest. Using a data-driven approach to compare the distribution of behaviourally distinct subsets of cells in a heterogeneous population from two donors, we found the greatest increase in migratory behaviour in cells with delayed or oscillatory motion for ICAM-1+IL-15 (trajectory C) and those with exploratory motion on VCAM-1+IL-15 (trajectory B) (Fig. 8B). Finally, we showed that VCAM-1 promotes exploratory migration characterized by frequent changes of behaviour, whereas ICAM-1 promotes oscillatory migration or delayed activation followed by steady migration.

## DISCUSSION

Traditional image analysis tools typically reduce rich datasets to population-level averages where tracking and morphology metrics are averaged across different conditions. Such analyses effectively average out heterogeneity generated by different subpopulations of cells. Here, we introduce cell plasticity analysis tool (cellPLATO), which is designed to embrace the heterogeneity present in biological microscopy data and apply cellPLATO to defining subpopulations of human NK cells migrating on integrin ligands in the presence or absence of IL-15.

### Comparison to existing tools

cellPLATO, a Python-based analysis tool, differentiates itself from existing phenomics tools like Traject3D (Freckmann et al., 2022), CellPhe (Wiggins et al., 2023), Heteromotility (Kimmel et al., 2018) and Lanternfish (Kimmel et al., 2021) in its approach to analysing cell behaviour in microscopy data. Unlike these tools, which often focus on either cell morphology or migration, cellPLATO integrates both using instantaneous, time-windowed and time-averaged measurements combined. The use of time windows enables the standardized comparison of cells between conditions and removes the biasing effect of the length of the cell track, mitigating tracking errors or experimental limitations such as the size of the field of view of imaging. The use of hand-engineered rather than learned features aids in human understanding of classes derived from dimensionally reduced space, can be added to in a modular fashion and works well with de-abstractification in this context. A focus on graphical de-abstractification aids in better understanding complex cell behaviours and enhances interpretability by allowing one to link population level heterogeneity with single cell behaviours mapped to the raw data. Unlike previous tools that measure cell behaviour (Freckmann et al., 2022; Wiggins et al., 2023), cellPLATO extracts cellular plasticity in the form of behaviour switching. Given that each cell in each cluster can be defined using 28 individual metrics, a cluster switch based on this definition is a rich descriptor, containing within it more information than single metrics and improving on previous methods to describe the relative dynamism of cell behaviour (Lee and Mace, 2017; Olofsson et al., 2014). Existing tools that work on phenomics data often use traditional *P*-value-based statistical testing only, often producing tiny *P*-values for insignificant biological changes (Gómez-de-Mariscal et al., 2021; Sullivan and Feinn, 2012). cellPLATO instead computes the and displays the effect size distribution between conditions overall or over time where an effect size distribution is calculated per timepoint (Goedhart, 2019 preprint). cellPLATO is designed to be used by individuals with limited coding expertise and takes three generalizable input formats (btrack, Usiigaci and Trackmate) to broaden access. Users minimally input a master folder path, pixel size and time resolution within a config file, then run a modular Jupyter notebook containing explanations of each step. Together, cellPLATO offers a new way to define clusters of behaviour, to understand the nature of cells in each cluster intuitively and to understand the switching between clusters of any population of cells.

### Patterns of cell shape and motility

Initially, we used the measurement and conditional comparison modules in cellPLATO to plot population-level data. We found that NK cells on ICAM-1 and VCAM1 react strongly and acutely to the addition of IL-15 by increasing their migration speed and cell area. Interestingly, NK cells on ICAM-1 underwent these increases more rapidly than those on VCAM-1. NK cells on ICAM-1 also had a much greater area with similar cell migration speed compared with those on VCAM-1, indicating that although NK cells increase their migration speed on both surfaces, they might be doing so using differing forms of migration. We subsequently used cellPLATO to investigate subpopulations of cells responding differently to IL-15 on each surface.

Using cellPLATO, we identified eight single timepoint behaviours (clusters) defined by their combinatorial morphology and migration characteristics. Although each of these clusters was present in cells on both surfaces, the proportion of cells in each cluster differed between ICAM-1 and VCAM-1. Specifically, ICAM-1 induced the prevalence of cells with larger areas, medium polarization and directed migration of medium speed. Conversely, VCAM-1 induced the prevalence of very fast cells with high polarization and less directed migration. These differences between modes of migration are consistent with ICAM-1-induced crawling behaviour in other lymphocytes (Nourshargh and Alon, 2014; Shannon et al., 2020) and VCAM-1-induced migration in which less of the cell body is in direct contact with the substrate (Dominguez and Hammer, 2014; Steiner et al., 2010). Both modes of migration have been previously visualized in human NK cells (Lee et al., 2020; Lee and Mace, 2017; Mace et al., 2016).

Although information about cell dynamics was extracted using clustering of single timepoint behaviours, we further sought to understand how cells could move between clusters throughout the time of imaging. Using trajectory analysis, we uncovered four trajectories of cell behaviour represented by different combinations of the single timepoint behaviours that were present in different proportions on ICAM-1 or VCAM-1. Cells adopted (1) stationary, semi-quiescent behaviour, (2) exploratory migratory behaviour, (3) delayed activation and then consistent migration behaviour or (4) oscillatory ‘stop and go’ confined migration type behaviour. The presence of stationary or delayed versus exploratory or oscillatory subsets suggests that some cells are pre-tuned to respond quickly to IL-15, whereas others need to upregulate or recruit components that are required for migration to the cell surface. Interestingly, despite population-level differences between donors in basic parameters of cell migration, such as cell speed, we found that these four modes of migration detected by trajectory analysis were conserved between different donors and were found in cells migrating in the presence or absence of IL-15. This reproducibility can be understood in three ways, namely (1) by visually comparing the cluster versus time plots for exemplars in Fig. 5 with those in Fig. 8, (2) by comparing the distributions of single timepoint behaviours in each trajectory (Figs 5G and 8G) and (3) comparing the cumulative cluster switches of each trajectory between Donor 1 (Fig. 5I) and Donors 2 and 3 (Fig. 8I). Although these four modes of migration were conserved, we found that the distribution of cells between these four trajectories was variable and differed between different donors and activation conditions.

The properties of the four trajectories of behaviour we identified in Donor 1 were reproducibly identified in Donors 2 and 3. Here, we collected a large amount of data, enabling us to show differences in proportion between conditions of less frequent behaviours, such as trajectory D, which nevertheless contained a minimum of 39 cell tracks based on 7024 timepoints. If certain behaviours are sufficiently infrequent or rare, they are more likely to be categorized as unclustered outliers. We therefore advocate for the collection of larger datasets to identify rare yet conserved behaviours of biological interest.

Finally, by imaging cells in the presence or absence of IL-15, we showed that IL-15 reduced the population of non-motile cells and increased other more migratory and exploratory populations. We saw acute increases in behavioural plasticity after the addition of IL-15, which were not present in cells that did not receive IL-15. We also describe subsets that respond relatively quickly or slowly to IL-15, confirming the acute effects of IL-15 on spontaneous migration in NK cells. We cannot rule out that cell division, known to be upregulated by IL-15 and not quantified here, generates additional cells tuned for one type of migration or another on either ICAM-1 or VCAM-1; however, the relatively short time of imaging would limit the number of cell divisions in this dataset. Taken together, these data demonstrate that both integrin ligand and IL-15 signalling can modulate the heterogeneous modes of migration of mature NK cells. In addition to demonstrating the utility of cellPLATO, these findings lay the foundation for future biological studies that will focus on better understanding the sources of heterogeneity between NK cell subpopulations.

### Limitations and next steps

Trajectory analysis allowed us to formally identify four subsets of behaviour in our enriched, yet heterogeneous, NK cell cultures, which we have described informally previously on stromal cell monolayer systems using manual and semi-automated tracking approaches (Lee et al., 2020; Lee and Mace, 2017; Mace et al., 2016; Martinez et al., 2022). The hardness of the glass surface (Lämmermann and Germain, 2014; Pham et al., 2016; Vesperini et al., 2021), the lack of integrin crosstalk produced by multiple ligands (Imhof et al., 1997; Kim and Hammer, 2019; Porter and Hogg, 1997), the lack of shear flow (Anderson et al., 2019) and the 2D nature of the system used here (Lämmermann and Germain, 2014) limited our biological interpretations to those specific to these surfaces. However, we provide new information about the acute response of subsets of mature NK cells to IL-15 on ICAM-1 and VCAM-1 on a stiff glass surface. Future studies could focus on linking these cell migration phenotypes with previously identified phenotypic subsets of mature NK cells. Our previous studies have identified differential modes of migration between NK cell developmental subsets (Mace et al., 2016), and these subsets also have differing expression of β1 and β2 integrins that mediate binding to ICAM-1 and VCAM-1 (Hegewisch-Solloa et al., 2021). Understanding how integrins, cytokine activation and developmental subsets interact to regulate motile scanning of NK cells is an ongoing area of interest.

Importantly, any analysis of cell tracking and segmentation data is limited by the quality of these upstream processes. Although cellPLATO does not itself contain a scoring method for segmentation or tracking, a debris filter is implemented to control for short or broken cell tracks and tiny objects wrongfully registered as cells, and time windowed analysis ensures track length does not bias the results. Currently, the centre of mass of each cell calculated during tracking is calculated based on segmentation of the cell membrane. Such tracking results in small shifts that register as cell displacement but could relate to a shape change, such as the extension of a lamellipodia. It would be useful to compare tracking using the centre of mass from cell nuclei with the cell membrane or cell outline, which should reduce these spurious displacements and might be highly relevant especially in the context of high magnification and/or 3D imaging.

It should also be noted that although cellPLATO is built to be simple to use for those new to coding, users are currently required to install the software locally. The first part of the cellPLATO workflow (feature extraction, UMAP, HDBSCAN, exemplar behaviour extraction at single timepoints and fingerprinting between conditions) has been reproduced in Google Colab (Jacquemet, 2023 preprint) providing browser-based access to remote computing resources, as well as using Docker – a containerized approach to provide a smooth local install process (Hidalgo-Cenalmor et al., 2024). Integration of cellPLATO with these platforms might be a future direction for the software. Its adaptable and modular format provides a good basis for community-driven development and usage, as it is amenable to the addition of new metrics that do not affect the downstream flow of analysis and can be implemented simply. In the future, cellPLATO will be extended to perform shape and motility analysis on data acquired at different magnifications and timescales and incorporate 3D measurements, network analysis, fluorescent reporters of cell state (Ghilardi et al., 2020; Zhang et al., 2023) and cell lineages or cell divisions (Bove et al., 2017; Ershov et al., 2022; Stirling et al., 2021; Tinevez et al., 2017; Ulicna et al., 2021). Although beyond the scope of the current study, we anticipate that cellPLATO will similarly be of value for analysing other subsets of migratory cells in 2D and 3D environments. We also anticipate that access to higher resolution cellular features in small subsets of cells placed within a population will be a powerful way to link molecular behaviours to populations of cells. Together, cellPLATO provides a user-friendly platform to analyse heterogeneity in complex cellular populations, is open source and adaptable to different inputs, and is under continued development. The current version of cellPLATO is available for download with complete instructions for use on GitHub (https://github.com/Michael-shannon/cellPLATO) with interactive Jupyter Notebooks provided.

## MATERIALS AND METHODS

### Primary human NK cell culture

Blood samples were collected in sodium heparin tubes (BD Vacutainer) from healthy donors at Columbia University Irving Medical Center. All samples were obtained with informed consent under guidelines established by the Declaration of Helsinki. Primary NK cells were isolated from peripheral blood by negative selection using RosetteSep human NK enrichment cocktail (Stemcell Technologies, 15065). Blood was layered onto a Ficoll–Paque (Thermo Fisher Scientific, 45-001-750) density gradient followed by centrifugation at 1200 ***g*** for 20 min with no brake. NK cells were collected from the density gradient interface and were placed into PBS at room temperature (RT), before being centrifuged again for 10 min at 300 ***g*** with a low brake. The cell pellet was then resuspended in 20 ml of warm cell culture medium R10 [RPMI 1640 (Thermo Fisher Scientific, 11875135), 10% heat-inactivated fetal bovine serum (FBS, Gemini, 900-108-500), 10 mM HEPES (Thermo Fisher Scientific, 15630130), 100 U/ml penicillin-streptomycin (Thermo Fisher Scientific, 15140163), 1×MEM non-essential amino acids (Thermo Fisher Scientific, 11140076), 1× sodium pyruvate (Fisher Scientific, 11360070), 1× L-glutamine (Glutamax, Thermo Fisher Scientific, 35050079)], prior to centrifugation at 120 ***g*** for 10 min with a low brake. Supernatant was discarded and cells were resuspended in R10 to a concentration of 2.5×10^6^ cells/ml. Cell solution was transferred to a 48-well plate and placed into an incubator at 37°C with 5% CO_2_ for 10 min prior to the addition of dyes and cytokines for microscopy or antibodies for flow cytometry as described below.

### Preparation of integrin ligand coated chamber slides for microscopy

A total of 40 µl of 0.01% poly-L-lysine in deionized H_2_O was added to each port of a µ-Slide VI 0.5 Glass Bottom chamber slide (Ibidi, 80607) which was incubated for 1 h at RT. Chambers were washed five times with 120 µl of PBS by addition to one port and removal at the opposite port. At the final wash, all liquid (160 µl) was removed before addition of integrin ligands – 160 µl of 1 µg/ml Fc-ICAM-1 (recombinant human ICAM-1/CD54 Fc Chimera Protein, CF, R&D, 720-IC-200) or 1 µg/ml Fc-VCAM-1 (recombinant human VCAM-1-Fc Chimera, Biolegend, 553706) were added to the chamber slide, which was then incubated at 37°C with 5% CO_2_ for 2 h. Migration medium (R10 with 1.2% FBS) was pre-equilibrated in the incubator at 37°C with 5% CO_2_ for 2 h prior to its use for washing the imaging plate by adding 120 µl of medium through one port and removing 120 µl of solution at the opposite port. This step was repeated twice before 120 µl of cells were added to the imaging chamber.

### Isolated NK cell preparation for fluorescence microscopy

NK cells were labelled with SPY650-DNA (Cytoskeleton, CY-SC501) and Cellbrite Steady 550 with Cellbrite Enhancer reagent (Biotium, 30107-T) by first making a 10× solution by diluting the three reagents 1:100 in R10 medium. IL-15 (Peprotech, 200-15) was added to this solution at 10× (0.1 µg/ml) for IL-15 conditions and the dye and cytokine solution was added to cells to a final concentration of 1× (10 ng/ml). The solution was mixed, and cells were incubated for 5 min at 37°C with 5% CO_2_. Following labelling, cells were directly added to the imaging chamber as described above.

### Timelapse confocal microscopy

µ-Slide channels were imaged using a confocal spinning disc microscope with a Yokogawa CSU W-1 spinning disc equipped with a 37°C incubator supplied with 5% CO_2_ (OKOlab). Prior to adding the sample to the microscope, the microscope, stage and insert were equilibrated for 2 h to enhance stability and reduce sample drift during imaging. The sample was subsequently equilibrated on the stage for 10 min. Tiled (2×2) and montaged time-lapse images of a single focal plane in multiple wells were obtained using a 20×0.8 NA air objective with a 40 s interval between time frames. Imaging was carried out using a 561 nm laser at 8% power with 120 ms integration time and a 640 nm laser at 9% power with 150 ms integration time. Imaging was performed for a total of 900 frames (600 min; 10 h) using an sCMOS camera (Teledyne Photometrics Prime 95B). To control for the potential toxicity of membrane and nuclear dyes and their exposure to laser light, brightfield imaging in cells free of dyes was carried out simultaneously to fluorescence imaging in a test experiment and no change in behaviour was observed.

### Image processing

2×2 tiled timelapse data was montaged in Slidebook 6.0 software (Intelligent Imaging Innovations) using a 5% image overlap before being exported as an image sequence of TIF files. The bioformats (Linkert et al., 2010) plugin in FIJI software (Schindelin et al., 2012) was used to open the sequence as a TIF stack. Images were cropped and then exported again using bioformats as an image sequence of a separate numbered TIF per timepoint. Each TIF contained two channels, where C1 was the membrane channel (SPY650-DNA) and C2 was the nuclear channel (Cellbrite Steady 550). We recommend using a file tree system as follows: all data stored within a master folder, which contains a separate folder for each condition (ICAM-1 or VCAM-1 in this experiment). Inside each conditional folder, there is a separate folder for each replicate (i.e. donor 1, donor 2 and donor 3). Timelapse movies are saved as image sequences inside each replicate folder.

### Cell segmentation

The human-in-the-loop feature of Cellpose 2.0 (Pachitariu and Stringer, 2022; Stringer et al., 2021) was used to retrain the existing neural network ‘Cyto2’, available in the model zoo. Ten example imaging frames were randomly selected from different conditions and replicates and were opened using the Cellpose GUI. A diameter of 28.6 was used with Cyto2 to successfully identify and segment most cells within the field of view. Each section of the field of view was examined and errors in segmentation were corrected manually by deleting the existing mask and repainting one or several manually. Errors occurred in tightly adjacent cells or in stretched out cells with both thick and thin regions. The corrected masks were used to retrain a new model called ‘Cyto2plus’ that was used for downstream analysis. To assess the quality of the segmentation, intersection over union (IoU) was used to compare manually annotated data with cyto2plus predicted data, yielding an IoU score of 0.89 (Fig. S8). The anaconda command line was used to batch segment all microscopy data within the master folder using Cellpose, communicating with an RTX 3090 GPU (NVIDIA) via PyTorch.

### Cell tracking

Tracking of the segmented cells was performed using btrack (Ulicna et al., 2021). Hyperparameters and priors for the Bayesian model were optimized by changing each parameter manually and checking the fidelity of example tracks versus manually tracked ground truths. More detail about model optimization can be found in the btrack documentation. btrack was run using Jupyter notebook (included with the cellPLATO package) in batch on the segmented masks in the master folder. Segmentations, *xy* coordinates and track IDs for each cell over time are saved into a separate h5 file per replicate.

### CellPLATO analysis

CellPLATO can be installed using pip, and full instructions are available on GitHub (https://github.com/Michael-shannon/cellPLATO). The software was built to be used by experimenters with no Python expertise and was tested and developed iteratively with users. Users input experimental parameters into a single config.py file including at a minimum: (1) the path to the master folder containing all data, (2) the names of the conditions to be included in the experiment, (3) the pixel size, and (4) the time resolution of the experiment. More advanced parameters include: (1) the size of the sliding time windows used to normalize some of the distance dependent metrics (i.e. cumulative length – in this case it was set to 5.3 min), (2) the filters placed on the data to exclude small/fast objects, such as debris from the analysis, and (3) cells that were tracked for fewer frames than the minimum time window. Here, only cells with an area of >50 µm^2^ and with at least eight timepoints were included. This choice of time window was based on an average NK cell migration speed of ∼3 µm/min and cumulative length of ∼16 µm per time window, reflecting the average length of two NK cells. Users should tune the size of the time window to their data. After completing and saving the config.py file, users can run the entire analysis using a Jupyter notebook. The notebook sequentially runs the measurement phase that uses scikit-image, as well as custom metrics (van der Walt et al., 2014), the dimensionality reduction, clustering, and de-abstractification phase, followed by trajectory analysis. Users can determine which features to use for UMAP and HDBSCAN; here, turn angle and Ripley's K were not used. For single timepoint clusters, UMAP nearest neighbours were set to 50, and HDBSCAN minimum cluster size was 500, with minimum samples at 300. For trajectories, UMAP nearest neighbours=15, HDBSCAN minimum cluster size=15 and minimum samples=10. A description of each of the metrics that is calculated is provided in Table S3. Cluster membership scoring is calculated using a silhouette score ranging from −1 to 1; a high score indicates that a given datapoint is well matched to its own cluster and poorly matched to other clusters. The dataset used for this article included three healthy adult blood donors. For each blood donor, imaging on different integrin ligands in the presence or absence of IL-15 was performed simultaneously to make time-based comparisons between conditions. Each imaging timelapse contained 900 frames of size 2030×2030 pixels. Total time for full analysis was 3 h using a mid-range CPU. Statistical testing between two non-parametric conditions was performed using Kruskal–Wallis testing. For multiple pairwise comparisons, Kruskal–Wallis was combined with Wilcoxon rank-sum testing with Bonferroni correction and calculation and plotting of the effect size distribution [also known as Plots of Differences (Goedhart, 2019 preprint)]. Crossing of the effect size distribution with the control line is an indication of statistical inconsequence (Sullivan and Feinn, 2012).

### Flow cytometry

NK cell enrichment was validated using a 5-color flow cytometry panel, confirming that >85% of enriched cells were CD56^+^CD3^−^CD19^−^CD14^−^ NK cells (Fig. S1). Cells were incubated with antibodies for CD3 (clone SK7, BV421, Biolegend, 1:200), CD14 (clone M5E2, BV421, Biolegend, 1:200), CD19 (clone HIB19, BV421, Biolegend, 1:200) and CD56 (clone HCD56, BV605, Biolegend, 1:100). A Zombie NIR Viability Dye was used for live/dead confirmation (1:200, Biolegend). Flow cytometry was performed on a Novocyte Penteon. All flow cytometry data analysis was performed with FlowJo software (BD Biosciences).

### Code availability

The latest version of cellPLATO can be found at https://github.com/Michael-shannon/cellPLATO. The version used for this paper can be found on Zenodo at doi:10.5281/zenodo.11267272.

## Supplementary Material

10.1242/joces.261887_sup1 Supplementary information
