# Supplementary material for: cellPLATO – an unsupervised method for identifying cell behaviour in heterogeneous cell trajectory data
Source: J Cell Sci. 2024 Jun 12;137(20):jcs261887. doi: 10.1242/jcs.261887 (PMC11213520; doi:10.1242/jcs.261887)
Supplement: Supplementary information [file joces-137-261887-s1.pdf]

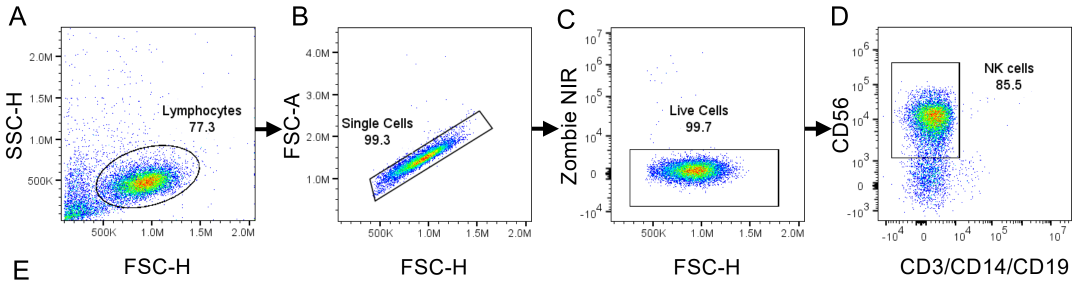

E

Fluorophore	Marker	Clone; Source; Dilution
BV421	CD3	SK7; Biolegend; 1:200
BV421	CD14	M5E2; Biolegend; 1:200
BV421	CD19	HIB19; Biolegend; 1:200
BV605	CD56	HCD56; Biolegend; 1:100

Fig. S1. Flow cytometry of enriched NK cells. NK cells were enriched from human peripheral blood as described in Methods. Flow cytometry analysis was used to confirm enrichment for NK cells. A) The lymphocyte population was defined by low granularity (SSC-H) and high forward scatter by height (FSC-H). B) Within this population, single cells were defined by height (FSC-H) being lower than area of each detection peak (FSC-A). C) Zombie NIR was used to gate on living cells within the single cell population. D) CD56 positive and CD3/CD14/CD19 negative cells were defined as NK cells. E) Table of fluorophores and antibody clones used for flow cytometry. Representative plots from one donor are shown.

Journal of Cell Science • Supplementary information

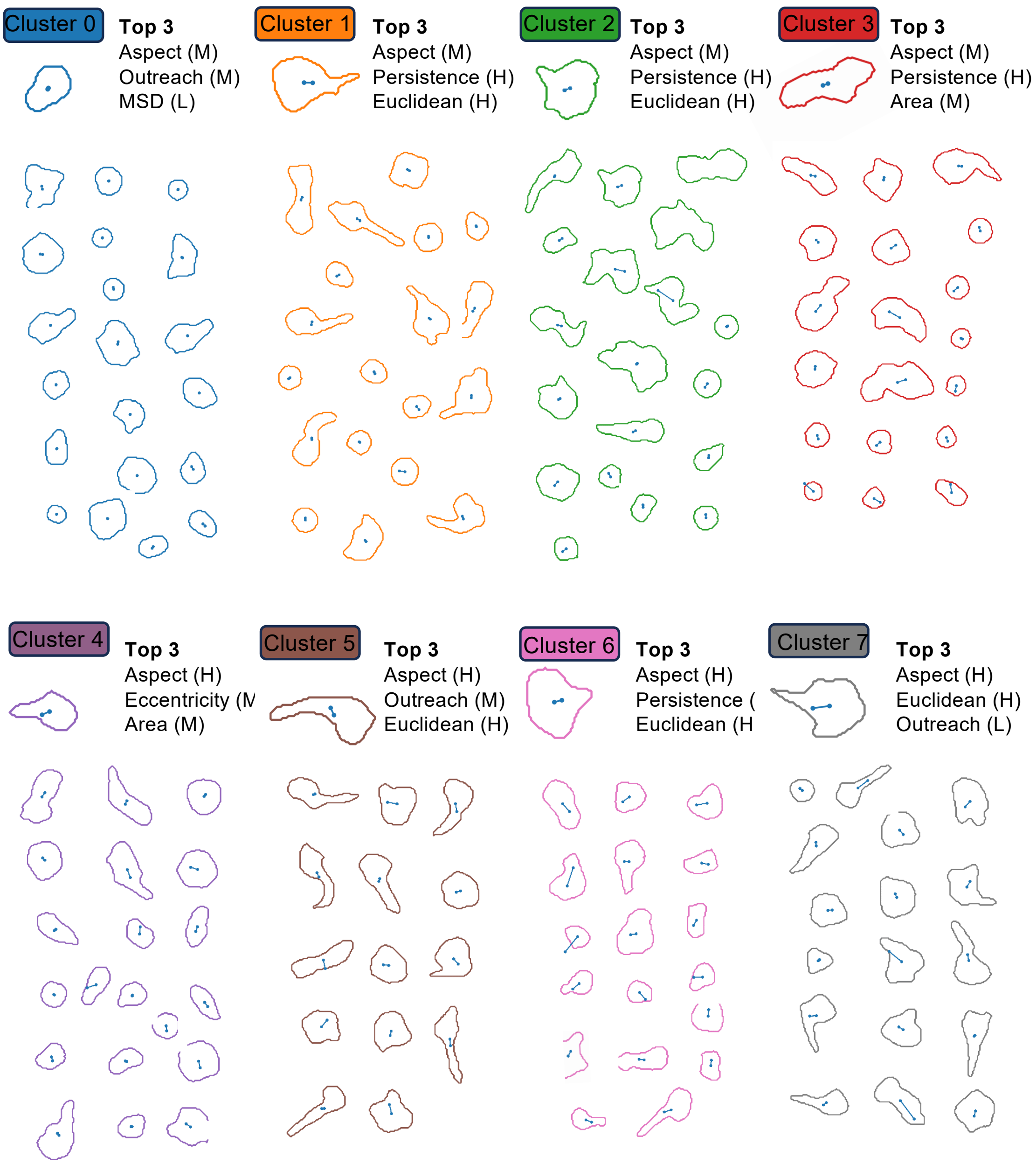

Fig. S2. Gallery of exemplar cells from each single timepoint behavioural cluster. Exemplar cell contour and tracks from clusters 0-7. The top 3 contributory metrics are labelled high (H), medium (M), or low (L). Donor 1 exemplars from n = 14,825 cell tracks from 1,036,092 individual datapoints.

Journal of Cell Science • Supplementary information

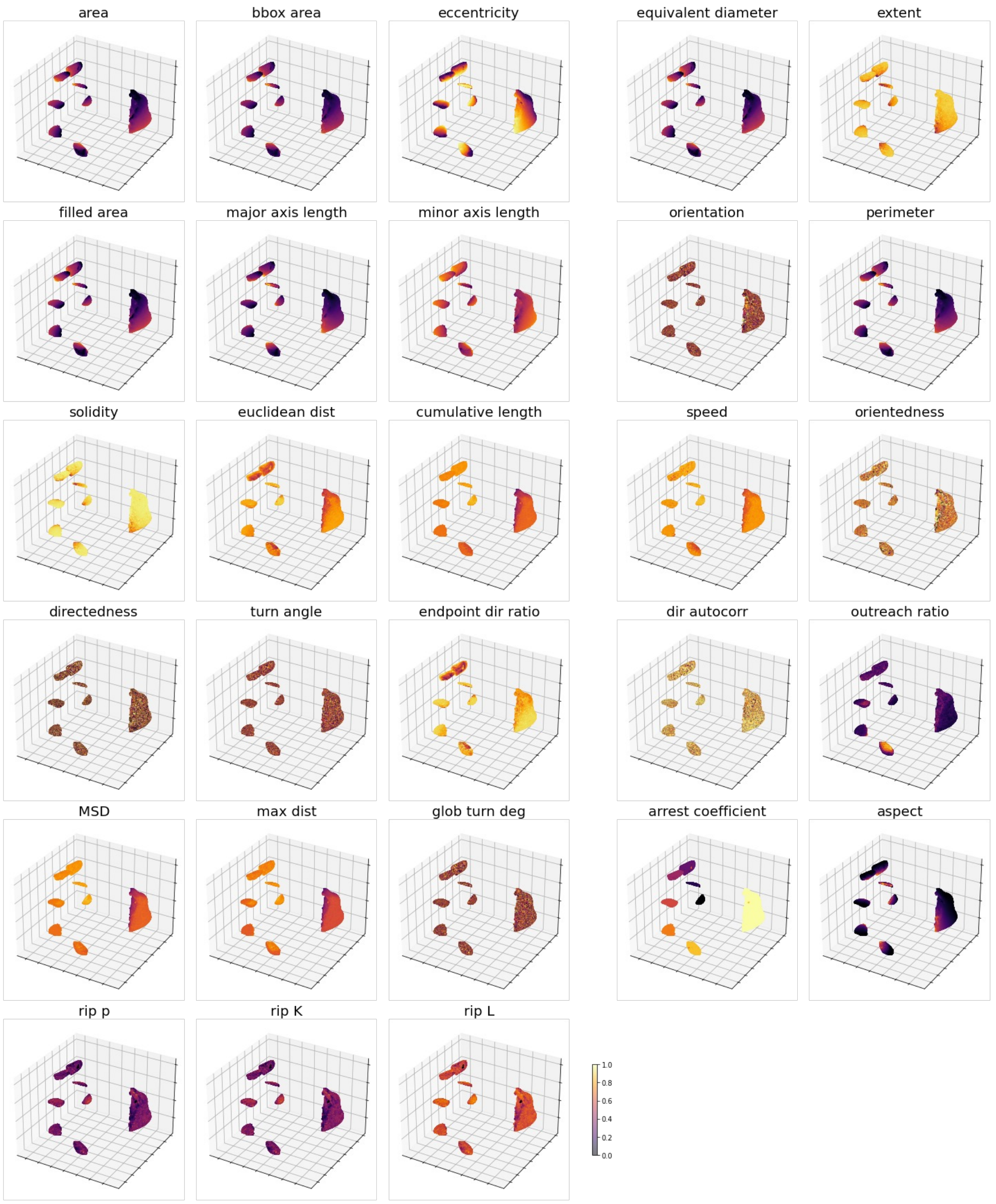

Fig. S3. UMAP plots coloured by contribution of each factor. Plots of each metric with scaling used for dimensionality reduction (log2 and minmax). Each datapoint coloured by magnitude of each factor. n=14,825 cells from Donor 1.

Journal of Cell Science • Supplementary information

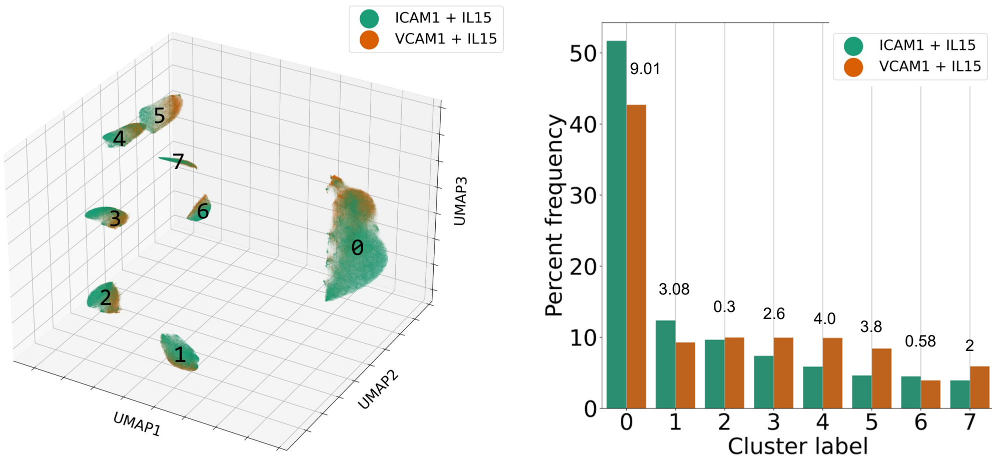

Fig. S4. NK cells occupy clusters differentially depending on their response to ICAM-1 or VCAM-1. A) 3D UMAP coloured by condition (ICAM-1, green; VCAM-1, orange). B) Percent occupancy of cells from each condition in each cluster, where the difference in percentage points between conditions within each cluster is printed above the bars. n = 14,825 cells from Donor 1.

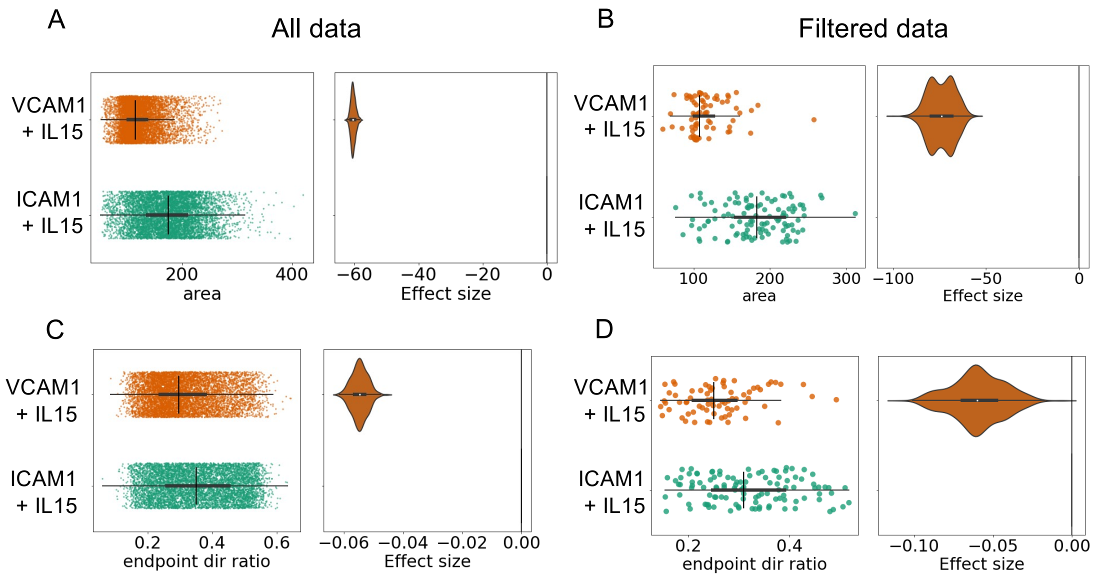

Fig. S5. Cell area and endpoint directionality ratio trends are maintained in filtered data compared with full data. Data was filtered to only include cell tracks between 200 to 220 timepoints in length to allows for sequence similarity measurement. A) Area of cells (plots of difference) for full data and B) filtered data. C) Endpoint directionality ratio (persistence length) for full data and D) filtered data. n= 14,825 cells in full data from Donor 1; 187 cells in filtered data.

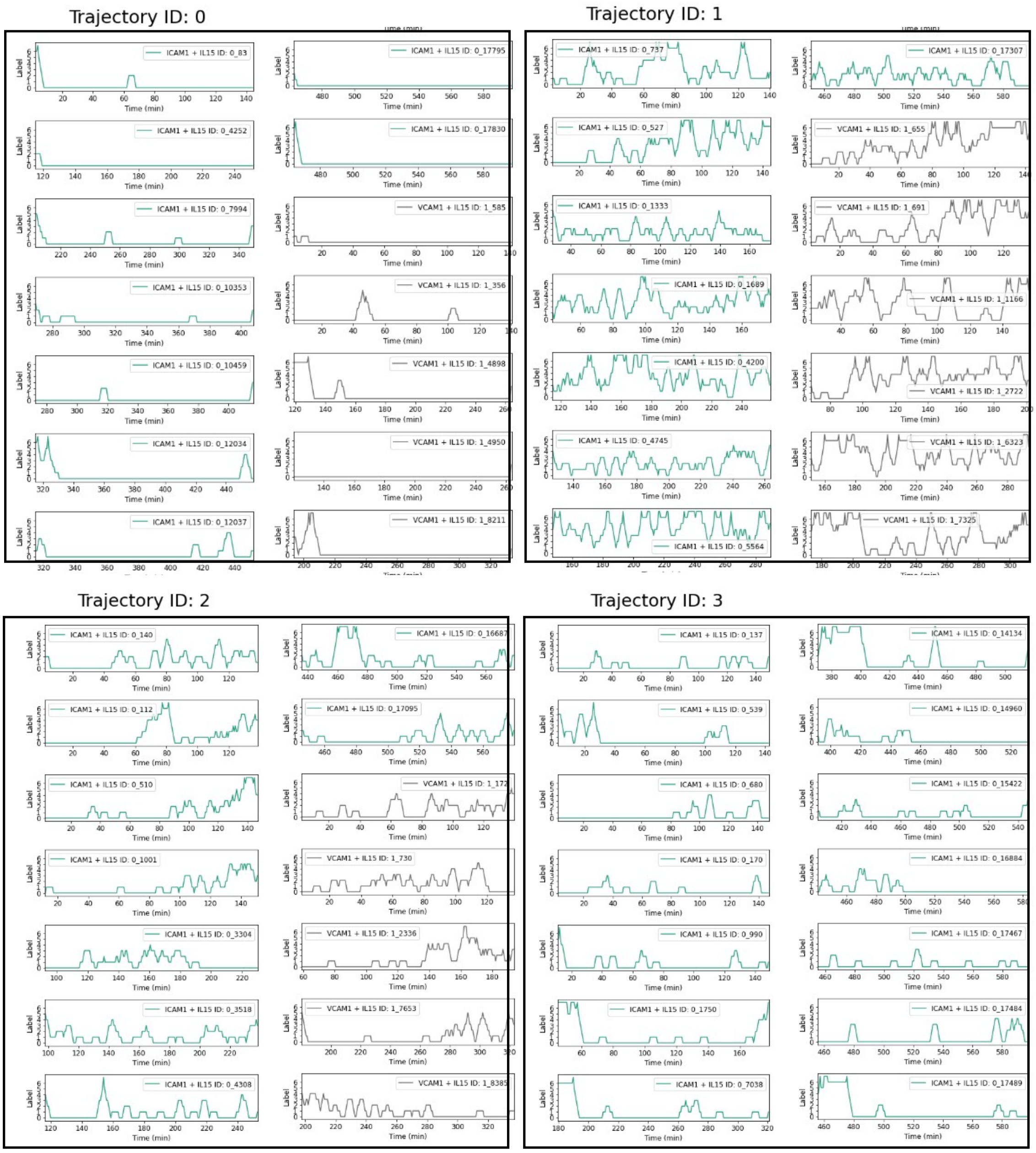

Fig. S6. Behavioural trajectory IDs as plots of behavioural cluster ID over time. Additional representative examples of cluster ID (label) over time for each trajectory ID (green, ICAM-1; grey, VCAM-1). Representative cells from n = 187 cells from Donor 1.

Journal of Cell Science • Supplementary information

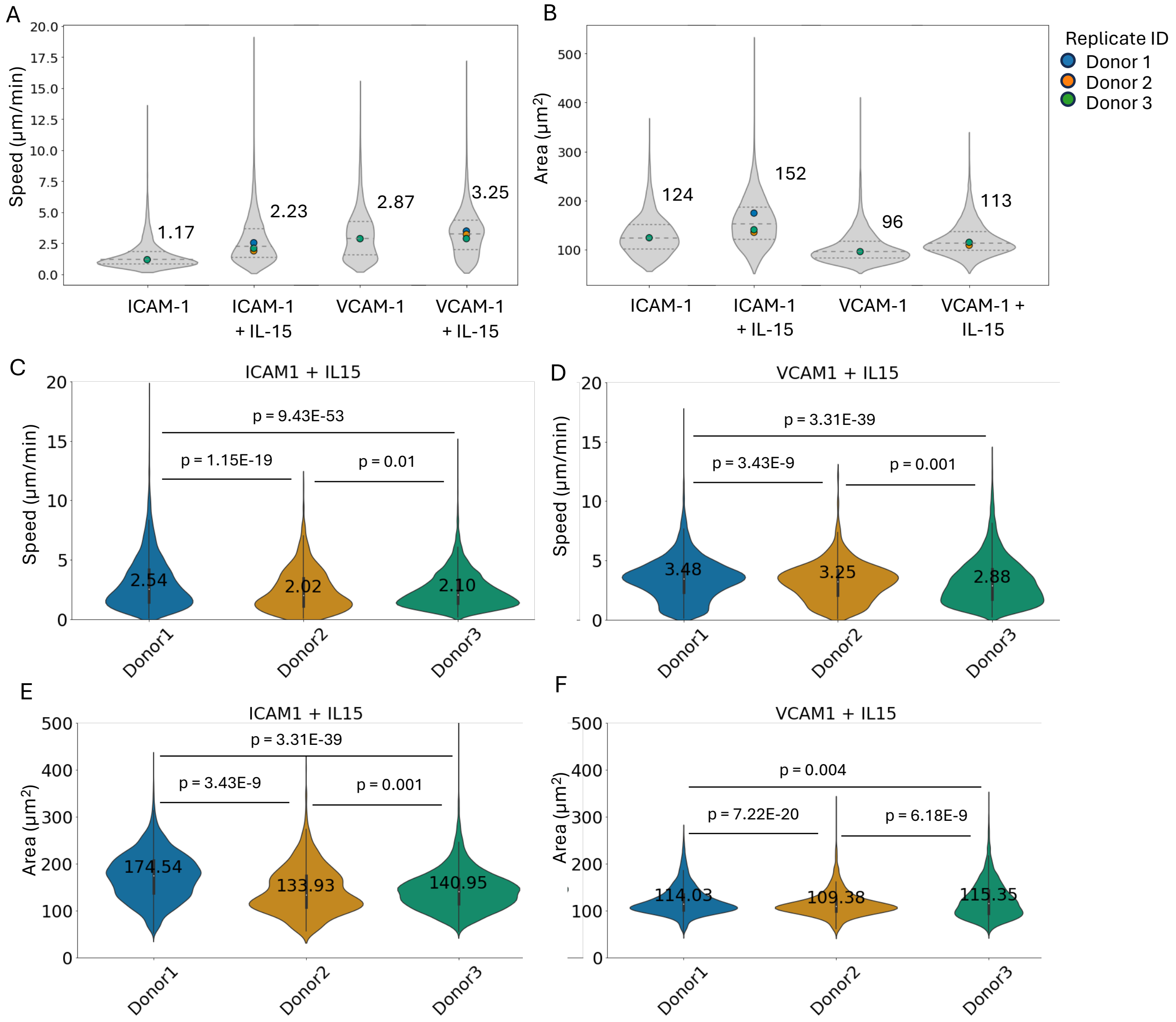

Fig. S7. Comparison of speed and area of NK cells between donors. A) Violin plot for speed showing distribution, median and IQR for pooled donors per condition. Coloured dots denote median value for each donor (donors 1, 2, and 3). B) Violin plot for area. C) Violin plots showing median speed of cells from each donor within ICAM1 + IL15 condition, and D) VCAM1 + IL15 condition. E) Area of each donor within ICAM1 + IL15 and F) VCAM + IL15. n = 47,815 cells analyzed in total, of which donor 1 = 14,825, 2 = 3150 and 3 = 29,840. Kruskal-Wallis followed by post-hoc Wilcoxon rank-sum test and Bonferroni correction was used to derive p values between donors and conditions. For plots A and D, statistical testing gave p<0.0001 for all pairwise comparisons.

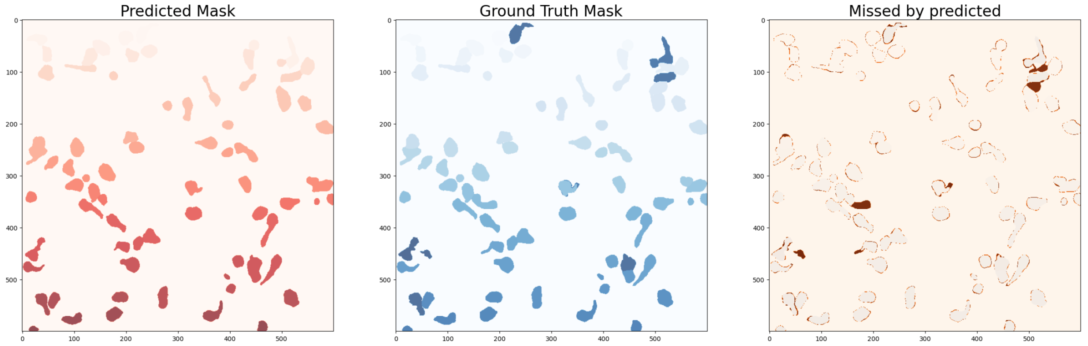

Fig. S8. Intersection of ground truth versus predicted masks from cellpose. A) Predicted masks derived from Cellpose. B) Ground truth mask derived from manual annotation. C) Pixels present in ground truth mask but not in predicted. Intersection over Union (IoU) Score: 0.8852.

Journal of Cell Science • Supplementary information

Table S1. Top 3 characteristics of cells belonging to 8 distinct behavioural clusters described by morphological and motility measurements. Peripheral blood derived IL-15 treated cells NK cells migrating on a surface of ICAM-1 or VCAM-1. Rows of table assigned by cluster ID. n = 14,825 cells from Donor 1.

Cluster ID	Characteristic 1	Characteristic 2	Characteristic 3	Morphology	Migration
**0**	Medium aspect ratio	Medium outreach ratio	Low MSD	Circular	Very low
**1**	Medium aspect ratio	High endpoint directionality ratio	High Euclidean distance	Slightly less circular	Medium
**2**	Medium aspect ratio	High endpoint directionality ratio	High Euclidean distance	Slightly less circular	Medium
**3**	Medium aspect ratio	High endpoint directionality ratio	Medium area	More spread, less circular	High
**4**	High aspect ratio	High eccentricity	Medium area	Polarized	Medium
**5**	High aspect ratio	Medium outreach ratio	High Euclidean distance	Polarized	Medium/high
**6**	High aspect ratio	High endpoint directionality ratio	High Euclidean distance	Polarized	Very straight and high
**7**	High aspect ratio	High Euclidean distance	Low outreach ratio	Polarized	Very fast, irregular

Table S2. Average characteristics for each of top three characteristics of cells belonging to 8 distinct behavioural clusters described by morphological and motility measurements. Peripheral blood derived IL-15 treated cells NK cells migrating on a surface of ICAM-1 or VCAM-1. n = 14,825 cells from Donor 1.

ClusterID	Metric	Median	Category
0	Aspect	1.1817	Medium
0	Outreach Ratio	0.2508	Medium
0	MSD	0.2553	Low
1	Aspect	1.3179	Medium
1	Endpoint Dir Ratio	0.3164	High
1	Euclidean Dist	2.663	High
2	Aspect	1.3422	Medium
2	Endpoint Dir Ratio	0.3017	High
2	Euclidean Dist	3.3373	High
3	Aspect	1.3731	Medium
3	Endpoint Dir Ratio	0.3077	High
3	Area	136.3985	Medium
4	Aspect	1.3944	High
4	Eccentricity	0.6969	Medium
4	Area	130.3428	Medium
5	Aspect	1.4072	High
5	Outreach Ratio	0.2372	Medium
5	Euclidean Dist	6.827	High
6	Aspect	1.3929	High
6	Endpoint Dir Ratio	0.5095	High
6	Euclidean Dist	14.8644	High
7	Aspect	1.4194	High
7	Euclidean Dist	9.674	High
7	Outreach Ratio	0.2206	Low

Journal of Cell Science • Supplementary information

Table S3. Description of metrics calculated for each cell at each timepoint. Instantaneous measurements were measured at individual frames for morphological characteristics and between frames for migration characteristics. Time windowed metrics are calculated using several grouped frames together to derive metrics over short timescales.

Metric	Definition	Instantaneous or Time Window
Speed	Distance traveled per unit time	Instantaneous
Area	Total area occupied by the object	Instantaneous
Bounding Box Area	Area of the smallest rectangle that encloses the object	Instantaneous
Eccentricity	Measure of how far the object is from being circular	Instantaneous
Equivalent Diameter	Diameter of a circle with the same area as the object	Instantaneous
Turn Angle	Angle turned by the object between adjacent frames	Instantaneous
Extent	Ratio: area of the object:area of smallest enclosing rectangle	Instantaneous
Filled Area	Area of the object excluding holes	Instantaneous
Major Axis Length	Length of major axis of ellipse with same second-moments as cell	Instantaneous
Minor Axis Length	Length of minor axis of ellipse with same second-moments as cell	Instantaneous
Orientation	Angle between x-axis and the major axis of the cell	Instantaneous
Perimeter	Perimeter of the object	Instantaneous
Solidity	Ratio: area of cell/area of its convex hull	Instantaneous
Aspect Ratio	Ratio: major/minor axis	Instantaneous
Ripleys K	Clustering of cells compared to random distribution within radius r	Instantaneous
Ripleys L	Normalizes K to interpret spatial patterns	Instantaneous
Euclidean Distance	Distance between start and end point	Time Window
Cumulative distance	Distance between each point summed over time	Time Window
Mean Squared Displacement	Mean squared displacement of the object	Time Window
Arrest Coefficient	Proportion of time spent below arrest threshold	Time Window
Max distance	Greatest distance travelled between adjacent frames within time window	Time Window
Outreach ratio	Ratio: max distance/cumulative distance	Time Window
Global turn degree	Total angle turned by the object from initial to current position	Time Window
Directional Autocorrelation	Sum of cosines of angles between frame-adjacent direction vectors	Time Window
Orientedness	Angle of current direction and projected direction (initial to endpoint)	Time Window
Endpoint directionality ratio	Ratio: euclidean/cumulative	Time Window
Directedness	How directed along the x axis the cell is	Time Window
Meandering Index	Deviation of cell trajectory from a straight line	Time Window

Journal of Cell Science • Supplementary information

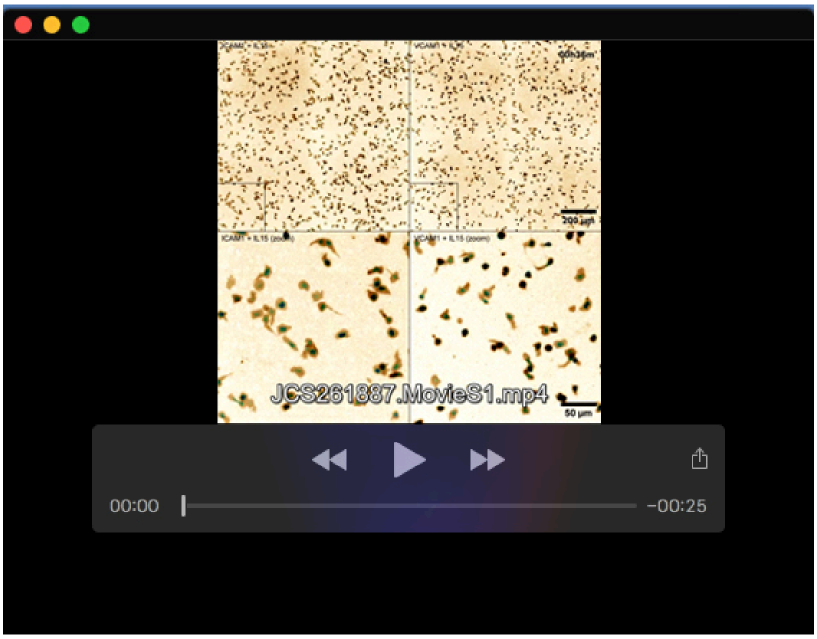

Movie 1. Timelapse micrographs of purified human NK cells migrating on ICAM1 or VCAM1 in the presence of IL15. Top panels show stitched fields of view of NK cells from Donor 1 migrating on ICAM1 (left) or VCAM1 (right). Bottom panels show zoomed regions denoted by black boxes for ICAM1 (left) and VCAM1 (right). Images are pseudocolored with yellow denoting cell cytoplasm labelled with Cellbrite Steady 550 and blue denoting nuclei labelled with SPY650-DNA. Micrographs are representative of several fields of view of cells from 3 donors imaged on each surface for 10 hours.

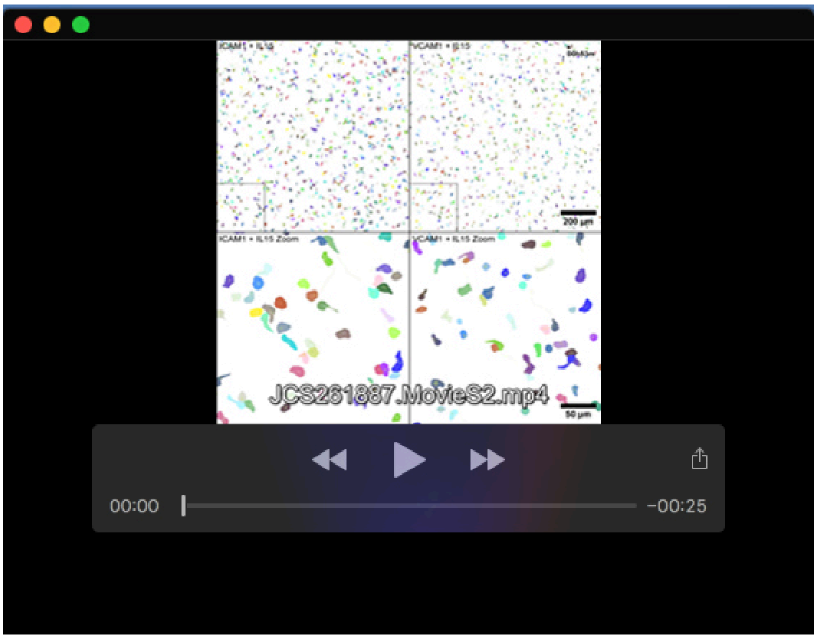

Movie 2. Segmentation and tracking of purified human NK cells migrating on ICAM1 or VCAM1 in the presence of IL15. Top panels show segmentation masks and tracks in stitched fields of view of NK cells from Donor 1 migrating on ICAM1 (left) or VCAM1 (right). Bottom panels show zoomed regions denoted by black boxes for ICAM1 (left) and VCAM1 (right). Segmented masks are arbitrarily colored per ID per frame, and tracks are colored by cell ID. Masks and tracks are representative of several fields of view from 3 donors imaged on each surface for 10 hours.

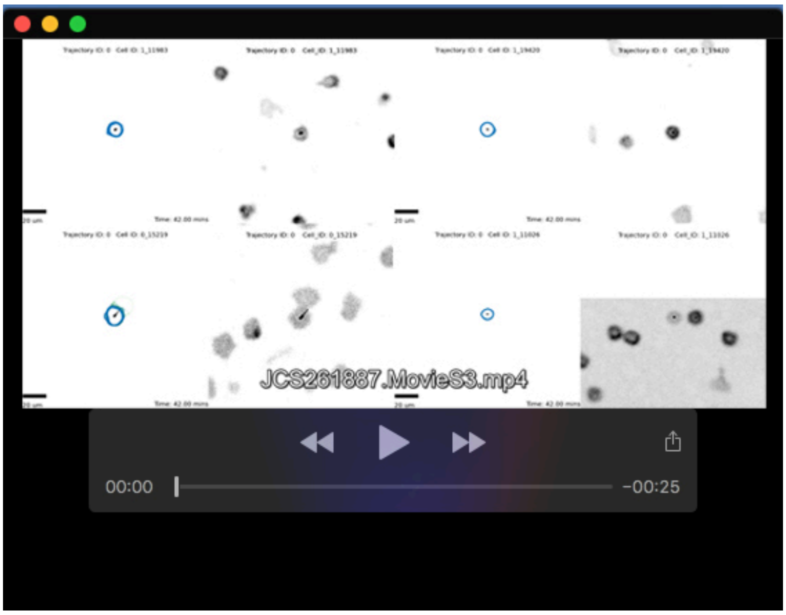

Movie 3. Trajectory cluster 0 animation of contour and track matched with raw image data. Several representative cells from trajectory cluster 0 extracted and displayed. For each cell, the left pane denotes their contours and tracks, where the colour of the contour denotes the single timepoint cluster ID and the black line represents the track. To provide context, the right pane shows the cell in the raw imaging data to which each contour/track relates.

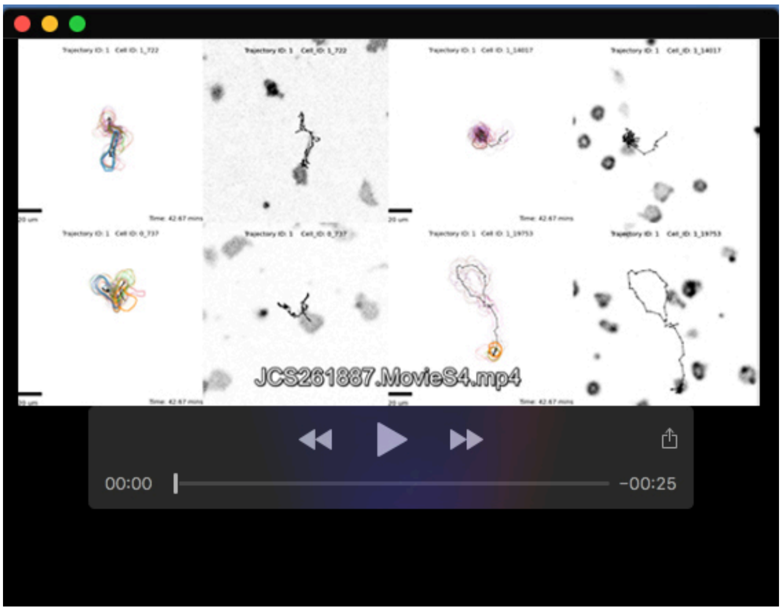

Movie 4. Trajectory cluster 1 animation of contour and track matched with raw image data. Several representative cells from trajectory cluster 1 extracted and displayed. For each cell, the left pane denotes their contours and tracks, where the colour of the contour denotes the single timepoint cluster ID and the black line represents the track. To provide context, the right pane shows the cell in the raw imaging data to which each contour/track relates.

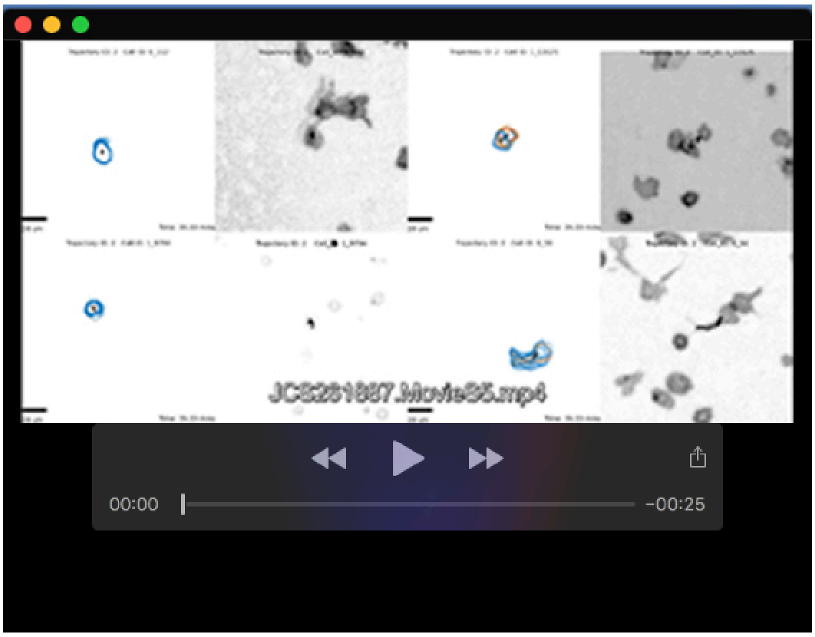

Movie 5. Trajectory cluster 2 animation of contour and track matched with raw image data. Several representative cells from trajectory cluster 2 extracted and displayed. For each cell, the left pane denotes their contours and tracks, where the colour of the contour denotes the single timepoint cluster ID and the black line represents the track. To provide context, the right pane shows the cell in the raw imaging data to which each contour/track relates.

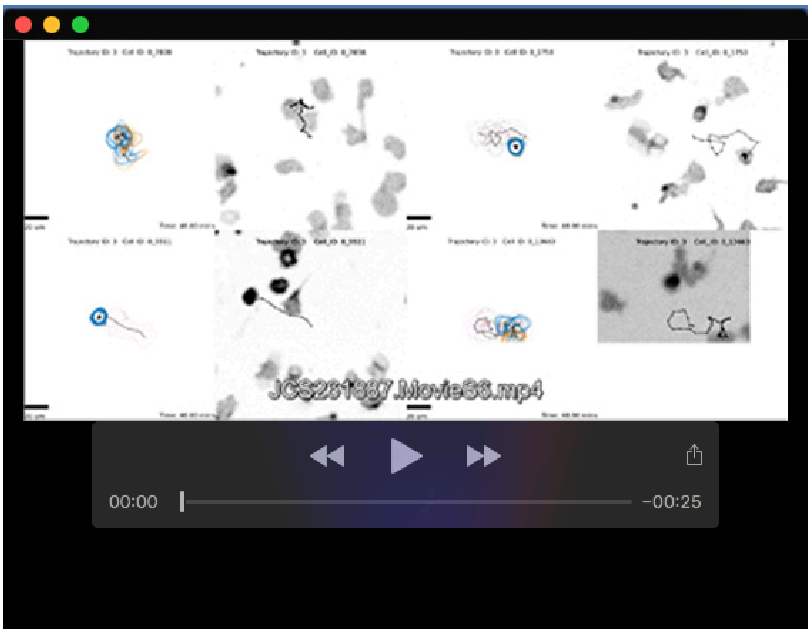

Movie 6. Trajectory cluster 3 animation of contour and track matched with raw image data. Several representative cells from trajectory cluster 3 extracted and displayed. For each cell, the left pane denotes their contours and tracks, where the colour of the contour denotes the single timepoint cluster ID and the black line represents the track. To provide context, the right pane shows the cell in the raw imaging data to which each contour/track relates.
